# Clinical and molecular response to tebentafusp in previously treated patients with metastatic uveal melanoma: a phase 2 trial

**DOI:** 10.1038/s41591-022-02015-7

**Published:** 2022-10-13

**Authors:** Richard D. Carvajal, Marcus O. Butler, Alexander N. Shoushtari, Jessica C. Hassel, Alexandra Ikeguchi, Leonel Hernandez-Aya, Paul Nathan, Omid Hamid, Josep M. Piulats, Matthew Rioth, Douglas B. Johnson, Jason J. Luke, Enrique Espinosa, Serge Leyvraz, Laura Collins, Howard M. Goodall, Koustubh Ranade, Chris Holland, Shaad E. Abdullah, Joseph J. Sacco, Takami Sato

**Affiliations:** 1 Herbert Irving Comprehensive Cancer Center, Columbia University Irving Medical Center, New York, NY USA; 2 Princess Margaret Cancer Centre, University of Toronto, Toronto, Ontario Canada; 3 Memorial Sloan Kettering Cancer Center, New York, NY USA; 4 Weill Cornell Medical College, New York, NY USA; 5 University Hospital Heidelberg, Heidelberg, Germany; 6 Stephenson Cancer Center, University of Oklahoma, Oklahoma City, OK USA; 7 Washington University School of Medicine, Saint Louis, MO USA; 8 Mount Vernon Cancer Centre – East and North Herts NHS Trust, Northwood, Middlesex UK; 9 The Angeles Clinic and Research Institute, a Cedars-Sinai Affiliate, Los Angeles, CA USA; 10 Institut Català d’Oncologia l’Hospitalet (Hospital Duran i Reynals), Hospitalet de Llobregat, Barcelona, Spain; 11 UC Cancer Center, University of Colorado, Aurora, CO USA; 12 Vanderbilt-Ingram Cancer Center, Vanderbilt University Medical Center, Nashville, TN USA; 13 UPMC Hillman Cancer Center, University of Pittsburgh, Pittsburgh, PA USA; 14 Hospital Universitario La Paz – CIBERONC, Madrid, Spain; 15 Charité Comprehensive Cancer Center, Charité – Universitätsmedizin Berlin, Berlin, Germany; 16 Immunocore, Abingdon-on-Thames, UK; 17 Immunocore, Rockville, MD USA; 18 Clatterbridge Cancer Center – NHS Foundation Trust, Wirral, UK; 19 University of Liverpool, Liverpool, UK; 20 Sidney Kimmel Cancer Center, Jefferson University, Philadelphia, PA USA

**Keywords:** Drug development, Translational research

## Abstract

In patients with previously treated metastatic uveal melanoma, the historical 1 year overall survival rate is 37% with a median overall survival of 7.8 months. We conducted a multicenter, single-arm, open-label phase 2 study of tebentafusp, a soluble T cell receptor bispecific (gp100×CD3), in 127 patients with treatment-refractory metastatic uveal melanoma (NCT02570308). The primary endpoint was the estimation of objective response rate based on RECIST (Response Evaluation Criteria in Solid Tumours) v1.1. Secondary objectives included safety, overall survival, progression-free survival and disease control rate. All patients had at least one treatment-related adverse event, with rash (87%), pyrexia (80%) and pruritus (67%) being the most common. Toxicity was mostly mild to moderate in severity but was greatly reduced in incidence and intensity after the initial three doses. Despite a low overall response rate of 5% (95% CI: 2–10%), the 1 year overall survival rate was 62% (95% CI: 53–70%) with a median overall survival of 16.8 months (95% CI: 12.9–21.3), suggesting benefit beyond traditional radiographic-based response criteria. In an exploratory analysis, early on-treatment reduction in circulating tumour DNA was strongly associated with overall survival, even in patients with radiographic progression. Our findings indicate that tebentafusp has promising clinical activity with an acceptable safety profile in patients with previously treated metastatic uveal melanoma, and data suggesting ctDNA as an early indicator of clinical benefit from tebentafusp need confirmation in a randomized trial.

## Abstract (web summary)

In a multicenter, single-arm, open-label phase 2 study of patients with previously treated metastatic uveal melanoma, treatment with tebentafusp, a soluble TCR bispecific (gp100xCD3) showed promising clinical activity with an acceptable safety profile.

## Main

Uveal melanoma is the most common primary eye tumour^1^ but it remains a rare condition, affecting fewer than 10 individuals per million^2,3^. Up to half of the patients with uveal melanoma will develop metastatic disease^4–6^, with the liver as the predominant site of distant spread^7^. Nearly all cases of uveal melanoma harbor one of four initiating oncogenic driver mutations in *GNAQ*, *GNA11*, *PLCB4* or *CYSLTR2* in a mutually independent fashion, as well as a secondary oncogenic event affecting *EIFA1X*, *BAP1* or genes encoding for spliceosome components, most commonly *SF3B1* (refs. ^8–10^). Uveal melanoma has one of the lowest mutational burdens of all malignancies, with approximately 0.5 mutations per megabase and a median of 32 coding mutations per tumour^11^, and is characterized by low expression of programmed cell death ligand 1 (PD-L1)^12^.

Tebentafusp, a first-in-class immune-mobilizing monoclonal T cell receptor (TCR) against cancer (ImmTAC), has been demonstrated in a phase 3 trial to improve overall survival in patients with previously untreated metastatic uveal melanoma when compared with investigator’s choice and represents the first therapy to demonstrate such a benefit in this disease^13^. The overall survival at 1 year was 73% in the tebentafusp group and 59% in the control group, with a hazard ratio (HR) for death of 0.51 (95% CI: 0.37–0.71, *P* < 0.001). These results are superior to those observed with targeted therapies such as selumetinib^14^ and immune checkpoint blockade. Two single-arm studies of combined ipilimumab and nivolumab in untreated and a mixed population of untreated and previously treated patients with metastatic uveal melanoma reported 1 year overall survival rates of 52% and 56% (refs. ^15,16^), respectively, which are similar to those reported in recent meta-analyses^17,18^.

Tebentafusp consists of a soluble affinity-enhanced TCR, specific for the gp100 peptide YLEPGPVTA–HLA-A*02:01 complexes presented on the surface of melanocytic cells, fused to an anti-CD3 single-chain variable fragment^19–22^. Once bound to their target gp100–HLA complex, polyclonal T cells are recruited and activated through CD3 ligation to release cytokines and cytolytic mediators against target cells^19–22^. In the first-in-human multicenter phase 1 study of tebentafusp, 3 of 15 (20%) evaluable patients with metastatic uveal melanoma achieved a partial response and 7 (47%) achieved stable disease, with an observed 1 year overall survival rate of 65% (ref. ^21^). A dose–response relationship was observed, with tebentafusp doses at or exceeding the maximum tolerated dose being associated with a greater response^23–25^. The phase 1 dose escalation portion of this phase 1/2 study (IMCgp100-102) was therefore designed to identify a higher and potentially more effective recommended phase 2 dose. Using a step-up dosing regimen to mitigate acute toxicity, a recommended phase 2 dose that was 36% higher than the maximum tolerated dose identified in the first-in-human trial was achieved^25–27^. An early analysis of safety and efficacy in these heavily pre-treated patients (*n* = 19) demonstrated a promising 1 year overall survival rate of 73%, despite a modest overall response rate of 11% (refs. ^25,26^), suggesting a decoupling of overall response rate as a surrogate for overall survival. This lack of correlation between overall response rate and overall survival was also observed in the tebentafusp cohort of the phase 3 trial in which the overall response rate was limited to 9% despite a very significant survival benefit^13^.

A similar but less pronounced disconnect between radiographic response and overall survival has been observed in other studies of immunotherapies^28,29^, prompting efforts to identify novel and effective surrogates for treatment benefit. Recent evidence indicates that a reduction in circulating tumour DNA is associated with clinical response to treatment in many cancer settings^30–34^ and can predict benefit to immunotherapy^30,35^. This is of particular importance given that activation of the immune system can lead to different kinetics of response to therapy^36,37^, and commonly used radiographic treatment response assessment criteria such as the Response Evaluation Criteria in Solid Tumours (RECIST)^38^ may not account for response patterns associated with immune activation, limiting their applicability in such settings.

The objective of the phase 2 expansion of IMCgp100-102 was to characterize the antitumour activity of tebentafusp in patients with previously treated metastatic uveal melanoma, a patient population distinct to that enrolled in the randomized phase 3 study, and to explore the association of early ctDNA dynamics with clinical outcomes in this setting.

## Results

### Study design

This open-label, international, phase 1/2 study (NCT02570308) included a phase 1 dose escalation as well as an expansion cohort and was subsequently expanded into a full phase 2 study. The results of the phase 1 dose escalation portion of this study have been recently reported^27^. Patients in the phase 2 study received weekly intravenous (i.v.) tebentafusp, initially at 20 μg on day 1, 30 μg on day 8, 68 μg on day 15 and then 68 μg i.v. once weekly thereafter as the recommended phase 2 dose, with the length of a treatment cycle defined as 4 weeks (28 days). Following at least the first three infusions, patients were observed for a minimum of 16 hours for monitoring of vital signs and, if necessary, provision of supportive care. After this induction period and provided that no grade 2 or higher hypotension was noted, the observation period for tebentafusp could be reduced to 30–60 minutes. Patients with an initial assessment of progressive disease according to RECIST v1.1 (ref. ^38^) could continue therapy beyond progressive disease provided that they did not have symptomatic progression requiring alternative therapy and the investigator believed that they were continuing to derive clinical benefit. After a patient was assessed as having RECIST progressive disease the treatment was continued until confirmation of immune-related progressive disease. This was defined as an additional ≥20% increase in tumour burden (that is, the sum of diameters of both target and new measurable lesions) as per the modified immune-related RECIST (irRECIST) criteria^39^, which were used to evaluate the response to and duration of treatment beyond progression.

The primary objective of the phase 2 study was to estimate the overall response rate based on RECIST v1.1. Secondary endpoints included safety, overall survival, progression-free survival, disease control rate (defined as the proportion of patients with either an objective response (that is, partial or complete response) or a best overall response of stable disease recorded at least 24 weeks (±1 week) after the date of the first dose of study drug), time to response, duration of response (defined as the time from the date of the first documented objective response (that is, partial or complete response) until the date of documented disease progression or death by any cause in the absence of disease progression), and the rate and duration of minor response, defined as a 10–29% reduction in the sum of the longest diameters of target lesions (Methods). Imaging-based endpoints were assessed by blinded, independent central review. For overall survival, patients who did not die were censored on the date on which they were last known to be alive. For progression-free survival and duration of response, patients who were known to be alive and without disease progression were censored at the date on which they were last known to be progression free. To assess potential predictors of efficacy of tebentafusp, changes in serum ctDNA levels were measured using a custom panel including mutations commonly found in uveal melanoma (GNAQ Q209L/P; GNA11 Q209L; SF3B1 K700E, R625L/H/C; PLCB4 D630N/Y/V; CYSLTR2 L129Q; and EIF1AX G15D) (Methods and Supplementary Table 4).

### Patients and treatment

**Extended Data Fig. 1:**
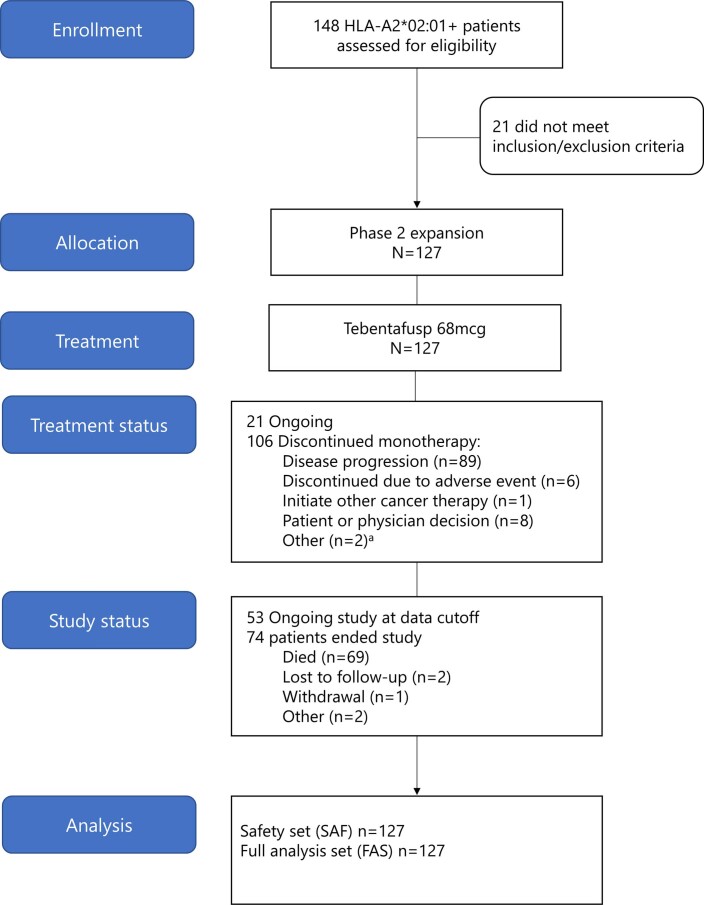
CONSORT flowchart for a single-arm, open-label, phase 2 clinical trial with tebentafusp in patients with metastatic uveal melanoma. All patients who received at least 1 full or partial dose of study drug are in the Safety Set. All patients assigned to treatment who received at least 1 full or partial dose of study drug are in the Full Analysis Set (FAS). ^a^One patient was classified as ‘Other – clinical disease progression’ rather than ‘Disease progression’ in error under reason for treatment discontinuation.

Of the 148 HLA-A*02:01-positive patients screened, 127 patients met the eligibility criteria and were enrolled between January and December 2017 in 26 study centers in five countries (Canada, Germany, Spain, United Kingdom and United States) (Extended Data Fig. 1). The median age was 61 years (range, 25–88 years) and 50% of patients were male (Table 1). The majority of patients (96%) had hepatic involvement. A total of 53% of patients had either American Joint Committee on Cancer (AJCC) M1b or M1c disease and 58% of patients had a baseline lactate dehydrogenase (LDH) level above the upper limit of normal (ULN). The median time from initial diagnosis to the development of metastatic disease was 3 years (range, 0–28 years) and the median and mean time since primary diagnosis to enrollment was 4.4 and 6.3 years (range, 1–28 years), respectively. All patients had received at least one prior line of therapy in the metastatic setting, with 34% receiving ≥2 lines of prior systemic (±liver-directed) therapy. More than two-thirds of patients (*n* = 90) received prior immune checkpoint inhibition, of whom 68% had primary resistance to treatment with a prior best response of progressive disease, and 32% relapsed, with a prior best response of at least stable disease, following treatment. At the time of data cut-off the median duration of study follow-up was 19.5 months (95% CI: 16–22.2 months).

**Table 1:** Demographics and baseline characteristics

	Phase 2	Baseline ctDNA (*n* = 94)	
	(*n* = 127)	< Median baseline ctDNA (*n* = 47)	≥ Median baseline ctDNA (*n* = 47)
Age (years), median (range)	61 (25–88)	62 (37–84)	60 (25–88)
Male sex, *n* (%)	63 (50)	21 (45)	29 (62)
ECOG performance status, *n* (%)			
0﻿	89 (70)	37 (79)	32 (68)
1	38 (30)	10 (21)	15 (32)
Time since primary diagnosis (years), median (range)	4.4 (1–28)	4.2 (1–23)	4.3 (1–28)
Time from primary diagnosis to metastatic disease (years), median (range)	3 (0–28)	3 (0–22)	3 (0–28)
No. of prior anti-cancer therapy regimens in the metastatic setting, median (range)	1 (1–5)	1 (1–4)	1 (1–4)
Previous anti-cancer therapy type in metastatic setting, *n* (%)			
Any	127 (100)	47 (100)	47 (100)
Systemic	105 (83)	38 (81)	38 (81)
Immunotherapy	92 (72)	35 (74)	30 (64)
Anti-PD1/Anti-PD-L1 monotherapy	52 (41)	24 (51)	12 (26)
Anti-CTLA4 monotherapy	8 (6)	3 (6)	2 (4)
Anti-CTLA4 and Anti-PD1	30 (24)	9 (19)	13 (28)
Other immunotherapy	5 (4)	2 (4)	3 (6)
Chemotherapy	14 (11)	4 (9)	6 (13)
Targeted therapy	10 (8)	2 (4)	8 (17)
Other	6 (5)	3 (6)	1 (2)
Radiotherapy^a^	10 (8)	4 (9)	2 (4)
Liver-directed therapy^a,b^	57 (45)	23 (49)	21 (45)
Surgery^a^	15 (12)	7 (15)	4 (9)
≥2 lines of prior systemic anti-cancer therapy^c^, *n* (%)	43 (34)	16 (34)	17 (36)
Previous anti-cancer therapy for primary disease, *n* (%)			
Any	125 (98)	46 (98)	47 (100)
Surgery^a^	41 (32)	12 (26)	14 (30)
Enucleation	40 (32)	12 (26)	14 (30)
Radiotherapy^a^	94 (74)	35 (74)	37 (79)
Brachytherapy	79 (62)	28 (60)	31 (66)
Systemic	11 (9)^d^	6 (13)	3 (6)
Elevated baseline LDH (>ULN), *n* (%)	74 (58)	18 (38)	41 (87)
Elevated baseline ALP (>ULN), *n* (%)	37 (29)	7 (15)	22 (47)
Baseline ALC ≥ 1.0 × 10^9^/l, *n* (%)	102 (80)	42 (89)	34 (72)
Metastasis location, *n* (%)			
Any hepatic	122 (97)	47 (100)	47 (100)
Hepatic only	47 (37)	22 (47)	13 (28)
Hepatic and extrahepatic	75 (59)	22 (47)	33 (70)
Extrahepatic only	4 (3)	2 (4)	1 (2)
Missing^e^	1 (1)	1 (2)	0 (0)
Largest liver lesion^f^			
M1a (≤3 cm)	45 (35)	22 (47)	8 (17)
M1b (>3 cm – ≤8 cm)	50 (39)	18 (38)	18 (38)
M1c (>8 cm)	17 (13)	2 (4)	15 (32)

ALC, absolute lymphocyte count; ALP, alkaline phosphatase; ECOG, Eastern Cooperative Oncology Group; ICR, independent central review; LDH, lactate dehydrogenase; ULN, upper limit of normal.

^a^Medically reviewed therapy types based on the coded term, reported term, electronic case report form therapy class, and reason for therapy. Also includes liver-directed chemotherapeutic agents.

^b^Liver-directed therapies include the following sub-categories: ablation, bland embolization, chemoembolization, immunoembolization, perfusion, radiation and radioembolization.

^c^Includes patients receiving liver-directed therapy in combination with systemic therapy.

^d^One patient received targeted therapy, two patients received immunotherapy (one received anti-PD1/L1 monotherapy and one received combination anti-CTLA4 and anti-PD1) and eight patients (6%) had missing information regarding the type of systemic therapy received in the primary setting.

^e^Measurable lesion by investigator was not confirmed by ICR.

^f^Liver metastases measurements based on ICR of target liver lesions only (AJCC Cancer Staging 8th edition).

Two-sided Fisher’s exact tests were conducted between categorical patient groups defined by baseline ctDNA and clinical characteristics.

Analysis of tumour biopsies (*n* = 63) showed that 61 (97%) had likely loss of one copy of *BAP1* (Methods), 23 (37%) had mutations in the gene encoding GNAQ, 26 (41%) in GNA11, three (5%) in CYSLTR2, one (2%) in PLCB4 and 11 (17%) in SF3B1. Mutations in *EIF1AX* were not detected in tumour biopsies.

All 127 patients (100%) received the study drug, of whom 21 (17%) remained on treatment at the time of data cut-off. The median duration of treatment was 5.5 months (range, 0–35 months). The primary reason for treatment discontinuation was disease progression (70%); six patients (5%) discontinued treatment due to an adverse event regardless of causality. At the data cut-off date, 53 patients (42%) remained in the study and 74 (58%) were reported to have ended the study. Death was the primary cause of study discontinuation (69/74; 93%). Nearly all of the deaths (67/69) were related to disease progression; the cause of death in two patients was listed as ‘other’, including cerebrovascular event in the setting of a fall in one, and clinical disease progression in the other. There were no deaths due to adverse events or caused by the study drug (Extended Data Fig. 1).

### Safety and adverse events

All patients had at least one treatment-related adverse event (Table 2 and Supplementary Table 2). The most frequently reported treatment-related adverse events of any grade could be classified as skin related, due to the targeting of gp100-positive melanocytes, or cytokine related, due to T cell activation, and included rash (87%), pyrexia (80%), pruritus (67%) and chills (64%). A total of 51 patients (40%) had a grade 3 event as their maximum grade, one-third of which were rash events (*n* = 20), and eight patients (6%) had a grade 4-related adverse event (hypotension and multiple organ dysfunction syndrome in one patient; lymphopenia; γ-glutamyltransferase increased; atrial fibrillation; amylase increased; hypophosphatemia; hypokalemia; and aspartate aminotransferase increased). A total of 25 patients (20%), 21 patients (17%) and four patients (3%) had treatment-related adverse events leading to hospitalization, dose interruptions and drug discontinuation, respectively. Following the 16 hour observation period after the first three doses, seven patients (6%) required an additional overnight stay due to a treatment-related adverse event. Treatment-related adverse events leading to discontinuation included atrial fibrillation and cytokine release syndrome, multiple organ dysfunction syndrome and cytokine release syndrome, left ventricular dysfunction, and dyspnea. There were no treatment-related deaths.

**Table 2:** Most common treatment-related adverse events^a^

	Any grade	Grade ≥ 3
	*n* (%)	*n* (%)
Any adverse event	127 (100)	59 (47)
Cytokine mediated		
CRS^b^	109 (86)	5 (4)
Pyrexia	101 (80)	5 (4)
Chills	81 (64)	1 (1)
Nausea	75 (59)	2 (2)
Fatigue	66 (52)	4 (3)
Hypotension	52 (41)	10 (8)
Vomiting	44 (35)	1 (1)
Headache	30 (24)	1 (1)
Skin related		
Rash^c^	111 (87)	20 (16)
Pruritus	85 (67)	5 (4)
Dry skin	50 (39)	1 (1)
Periorbital edema	34 (27)	0
Edema peripheral	33 (26)	1 (1)
Hair color changes	32 (25)	0
Skin exfoliation	28 (22)	0

^a^Treatment-related adverse events that were present in at least 20% of patients at any grade.

^b^Cytokine release syndrome (CRS) was graded according to the 2019 ASTCT Consensus Grading for CRS.

^c^Rash is a composite term for a list of skin toxicities of any grade (Supplementary Table 1).

**Fig. 1:**
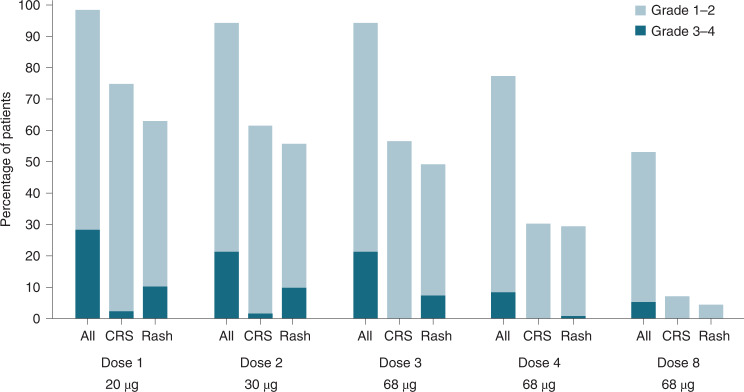
Incidence and severity of treatment-related adverse events after the initial tebentafusp doses. Percentage of treated patients with grade 1–2 grade 3–4 treatment-related adverse events after each dose of tebentafusp. A total of 127 patients received dose 1, 122 received dose 2, 122 received dose 3, 119 received dose 4 and 113 received dose 8. There were no treatment-related deaths. CRS, cytokine release syndrome.

Consistent with the phase 1 studies, adverse events related to tebentafusp, including rash, generally occurred early in the course of treatment and reduced in incidence and severity with repeated dosing, with ~65% of patients having rash from weeks 1 to 3 and 23% in week 8 (Fig. 1). Patients with symptomatic rash were generally managed successfully with antihistamine and topical corticosteroid therapy, and no patient discontinued treatment due to rash.

Cytokine release syndrome is a common adverse event with T cell engaging therapies and 109 patients (86%) had cytokine release syndrome based on American Society for Transplantation and Cellular Therapy (ASTCT) consensus grading criteria^40^. Most patients had either grade 1 (33%) or grade 2 (49%) cytokine release syndrome as their maximum grade. Very few patients had grade 3 (3.1%) or 4 (0.8%) events. Two patients had cytokine release syndrome events leading to discontinuation, including a patient who had a serious adverse event of grade 3 cytokine release syndrome on cycle 1 day 1 (C1D1) with a concurrent serious adverse event of grade 4 atrial fibrillation. The patient was treated with i.v. fluids, paracetamol, i.v. methylprednisolone, and oxygen. The cytokine release syndrome resolved the next day and the study drug was discontinued due to the event of atrial fibrillation. The second patient had grade 4 cytokine release syndrome on C1D1 with concurrent adverse events of grade 1 pyrexia, grade 4 hypotension and grade 4 multiple organ dysfunction. The patient received i.v. fluids followed by i.v. steroids, tocilizumab and vasopressors and was intubated for respiratory support. The event of cytokine release syndrome resolved 2 days later, and the study drug was discontinued due to the event of multiple organ dysfunction.

The onset of cytokine release syndrome, based on an increase in body temperature, generally began within 8–10 hours following administration and, as with other treatment-related adverse events, most events occurred following the first three doses, with a marked reduction in the incidence and severity of cytokine release syndrome thereafter (Fig. 1). All five grade 3 and 4 episodes occurred following one of the initial two doses, during the step-up dosing regimen. Patients were generally treated with antipyretics (*n* = 96, 88%), i.v. fluids (*n* = 48, 44%) and/or systemic glucocorticoids (*n* = 28, 26%). Supplemental oxygen (*n* = 9, 8%), vasopressors (*n* = 2, 2%) and tocilizumab (*n* = 2, 2%) were less frequently used to manage more severe cases.

### Tumour response and progression-free survival

**Extended Data Fig. 2:**
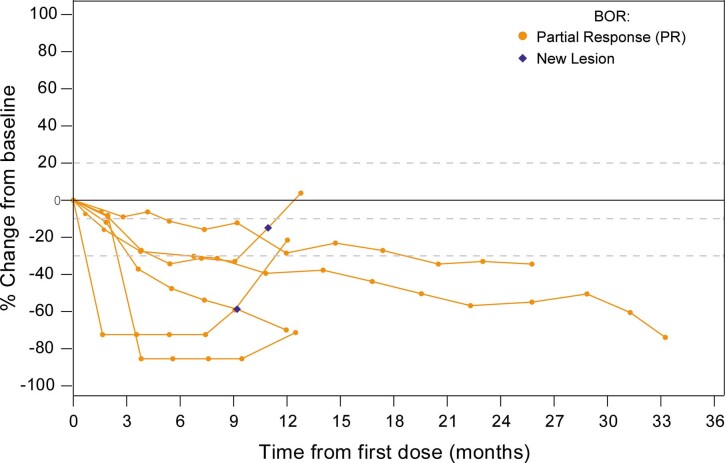
Tumour size change from baseline over time for patients with best overall response of partial response. Spider plots showing change in tumour size over time for individual patients with a best overall response of partial response (PR) (n = 6). BOR = best overall response.

The primary endpoint of overall response rate as per RECIST v1.1 by independent central review was 5% (95% CI: 2–10%) (Table 3), with six patients achieving a partial response (Extended Data Fig. 2). Of these six patients, three had ongoing responses for >6 months, one had an ongoing response for >12 months and one patient was censored at 5.3 months. The median duration of response was 8.7 months (95% CI: 5.6–24.5 months) and the median time to response was 4.6 months, with response times ranging from 1.6 to 20.5 months.

**Table 3:** Best overall RECIST response rate

	Tebentafusp (*n* = 127)
	*n* (%), (95% CI)
Objective response rate	6 (5), (2–10%)
Partial response	6 (5)
Stable disease^a^	57 (45)
Minor response^b^	8 (6)
Progressive disease	60 (47)
Non-evaluable/Not applicable	4 (3)
Disease control rate at ≥24 weeks (CR/PR/SD)	29 (23), (16–31%)

Tumour assessment was based on RECIST v1.1 by independent central review.

^a^Stable disease ≥8 weeks

^b^A minor response was defined as a reduction from baseline in the sum of longest diameters (or short axis for lymph nodes) of target lesions (mm) of 10–29%, where non-target lesion response was not unequivocal progression, and no new lesions were present. Confirmation was required after ≥4 weeks.

CR, complete response; PR, partial response; SD, stable disease.

**Extended Data Fig. 3:**
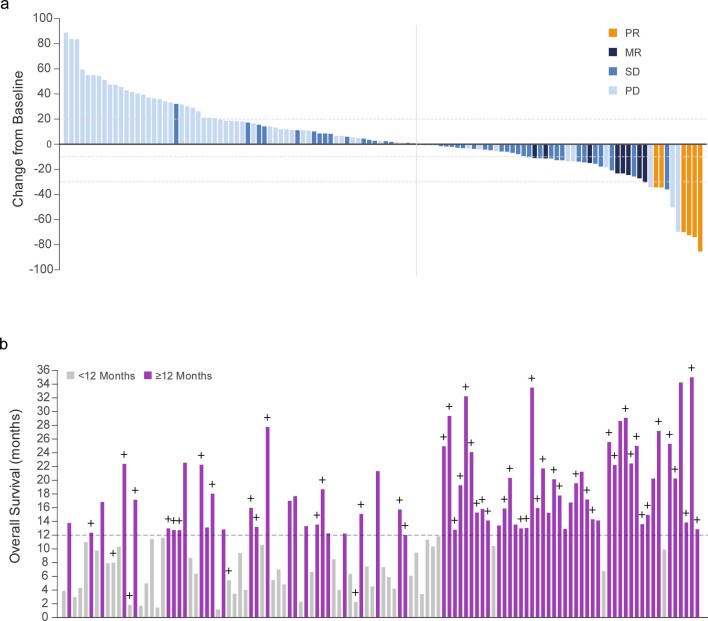
Clinical activity of tebentafusp. **(a)** Waterfall plot showing the best change in tumour size (n = 116). 44% of patients had tumour reduction at any time. Tumour size was measured as the sum of longest diameters or short axis of the target lesions according to RECIST v1.1 by Independent Central Review. Best percent change in target lesion size was the maximum percent reduction from baseline or the minimum percent increase from baseline (in the absence of a reduction), up until disease progression or starting subsequent alternative cancer therapy. Complete response, partial response or minor response required confirmation at least 4 weeks later. Reference lines at 20%, -10%, and -30% mark target lesion response criteria for disease progression (PD), minor response (MR), and partial response (PR), respectively. SD, stable disease. **(b)** Overall survival in months is plotted for each patient (n = 116). 61.2% of patients survived more than 12 months. + denotes censored. (A-B) Only patients with at least one evaluable post-baseline target lesion scan were included. Eleven patients overall were not included due to non-measurable disease at baseline or no evaluable post-baseline target lesion scans. Evaluable post-baseline scans must be on or prior to disease progression or starting subsequent alternative cancer therapy to be considered.

Of the 127 patients, 57 (45%) achieved stable disease at ≥8 weeks. The disease control rate was 32% (*n* = 40; 95% CI: 24–40%) at ≥16 weeks and 23% (*n* = 29; 95% CI: 16–31%) at ≥24 weeks. Any tumour shrinkage of target lesions was observed in 44% (*n* = 51) of evaluable patients (*n* = 116), including 10 of 55 patients (18%) with a best RECIST response of progressive disease (Extended Data Fig. 3a), consistent with radiographic pseudoprogression.

**Extended Data Fig. 4:**
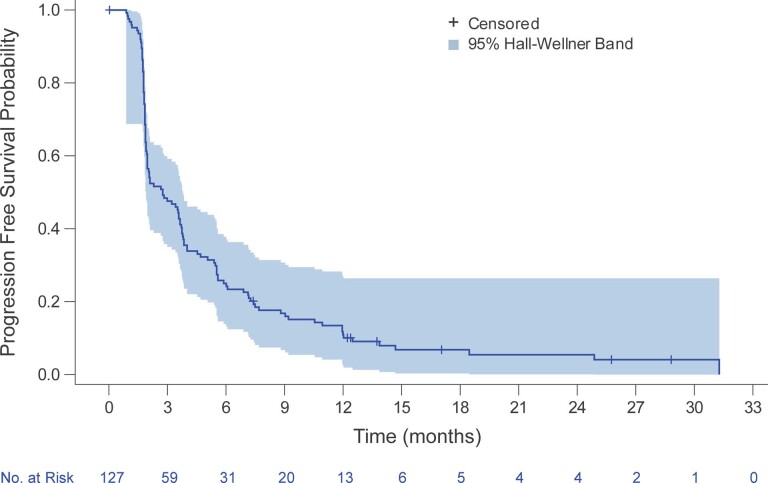
Progression-Free Survival. Kaplan–Meier estimate of progression-free survival for patients treated with tebentafusp. Tick marks represent patients who were known to be alive and without disease progression as assessed per Response Evaluation Criteria in Solid Tumours, version 1.1, by blinded, independent, central radiologic review. The median progression-free survival was 2.76 months (95% CI: 2-3.7) and was estimated by the Kaplan–Meier method.

**Extended Data Fig. 5:**
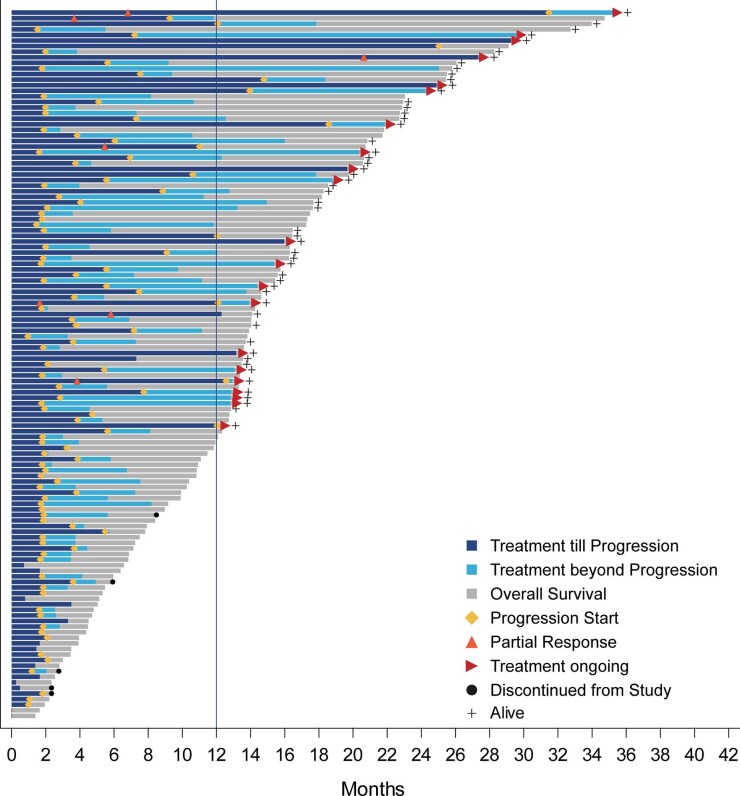
Swimlane plot of overall survival and treatment beyond progression. Swimlane plot showing overall survival, duration of treatment till progression and duration of treatment beyond initial progression (N = 127). The median duration of treatment beyond progression was 2.9 months (range: 0-23.1 months; n = 90).

The median progression-free survival was 2.8 months (95% CI: 2–3.6 months). The estimated progression-free survival rates were 25% (95% CI: 18–33%) at 6 months and 11% (95% CI: 6–17%) at 12 months (Extended Data Fig. 4). A total of 90 patients (71%) were treated beyond initial disease progression, with a median duration of treatment following confirmation of RECIST progressive disease of 2.9 months (range, 0–23.1 months; Extended Data Fig. 5). Of these patients, six (7%) achieved immune-related stable disease and 69 (77%) had confirmed immune-related progressive disease, per modified irRECIST as assessed by blinded, independent central review.

### Overall survival

**Fig. 2:**
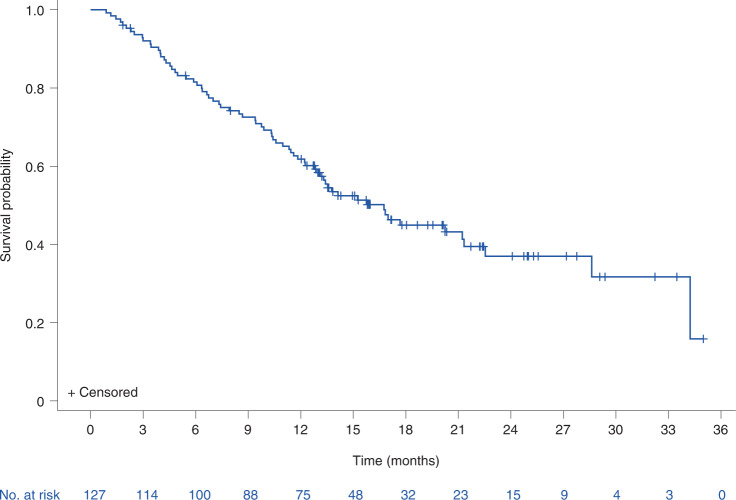
Overall survival for tebentafusp-treated patients with metastatic uveal melanoma. Kaplan–Meier plot of overall survival (*n* = 127). Events are deaths due to any cause. Patients not known to have died at the time of analysis are censored (+). The median overall survival was 16.8 months (95% CI: 12.9–21.3 months) with a 1 year overall survival rate of 62% (95% CI: 53–70%). The 1 year overall survival rate, median overall survival and corresponding two-sided, 95% confidence intervals were estimated using the Kaplan–Meier method.

**Extended Data Fig. 6:**
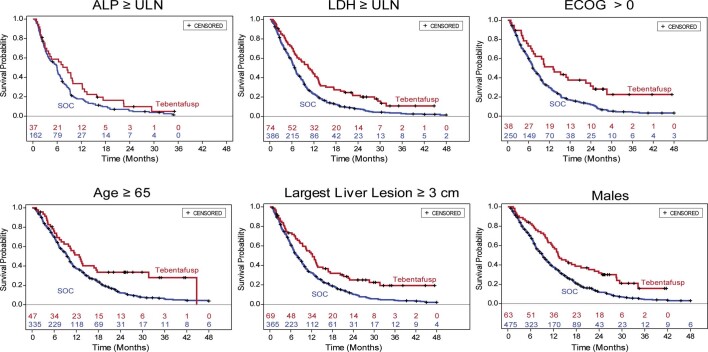
Subgroup analysis of OS in the six major prognostic categories from this current study compared to a recent meta-analysis. Digitized overlay of Kaplan–Meier estimates of overall survival for patients treated with tebentafusp in this study compared to curves from patients with the same negative baseline prognostic factor from a meta-analysis^17^ for ALP ≥ ULN, LDH ≥ ULN, ECOG > 0, Age ≥ 65, largest liver lesion ≥ 3 cm and male sex.

**Extended Data Fig. 7:**
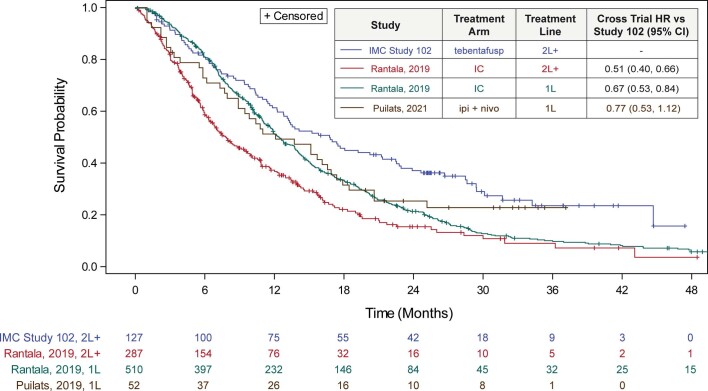
Tebentafusp OS in 2 L + population compared to meta-analysis and combination checkpoint inhibitors. Digitized overlay of Kaplan–Meier estimates of overall survival for patients treated with tebentafusp in this study compared to 2 L + and 1 L populations from a meta-analysis (Rantala et al. 2019)18 and 1 L patients treated with combination ipilimumab and nivolumab in a single-arm phase 2 study (Piulats et al. 2021)16. Hazard ratios and 95% CIs calculated from a univariate Cox proportional hazards model. 1 L = first line, 2 L + = second line plus, IC = investigator’s choice, ipi = ipilimumab, nivo = nivolumab, OS = overall survival.

**Extended Data Fig. 8:**
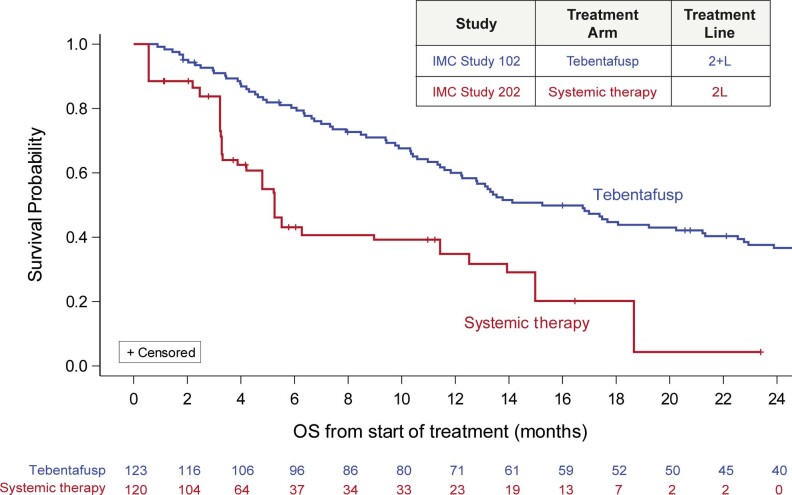
IPTW-Adjusted Kaplan–Meier Estimates of OS by Treatment Group. Adjusted product-limit survival estimates with number of subjects at risk comparing OS from start of treatment of tebentafusp from this study (Study 102) (blue, n = 123) and Study 202 subsequent systemic therapy (red, n = 120). Using Propensity Scoring methods (IPTW), control patients in the IMCgp100-202 phase 3 study who received standard of care 2 L therapies were weighted so that, overall, they are similar with respect to their baseline prognostic factors to the tebentafusp-treated patients from this study (102). The primary analysis involving 2 L + patients from Study 102 compared to control patients in Study 202 who received any systemic therapy as their 2 L treatment resulted in an HR (95% CI) of 0.40 (0.29, 0.55). The hazard ratio was derived from a weighted Cox proportional hazards model and the 95% confidence interval was derived using robust sandwich estimation from the weighted Cox model. IPTW = inverse probability of treatment weights.

With a median duration of study follow-up of 19.5 months (95% CI: 16–22.2 months), the median overall survival was 16.8 months (95% CI: 12.9–21.3 months) in this patient population with previously treated metastatic uveal melanoma. The estimated 1 year and 2 year overall survival rates were 62% (95% CI: 53–70%) (Fig. 2) and 37% (95% CI: 27–48%), respectively. Longer survival was associated with development of any tumour shrinkage, including partial response by RECIST criteria, with most patients (86%) with tebentafusp-induced tumour shrinkage alive at 12 months (Extended Data Fig. 3b). Even among patients with tumour growth (≥20% increase from baseline) as best change on treatment, 43% (12/28) were alive at 12 months. In predefined subgroup analyses, the overall survival at 1 year was 51% in patients ≥65 years of age, 45% for patients with elevated LDH at baseline, and 75%, 60% and 25% in patients with largest target liver metastasis at baseline of ≤3 cm (M1a), >3 cm to ≤8 cm (M1b) and >8 cm (M1c), respectively. In patients who had previously relapsed (best overall response of complete response, partial response or stable disease on prior therapy) following immunological checkpoint inhibition, 1 year overall survival was 76% (95% CI: 56–88%) compared with 60% (95% CI: 46–71%) in patients who were refractory (best overall response of progressive disease on prior therapy) to prior checkpoint inhibition (Supplementary Table 3). This survival appears superior when compared with similar populations from meta-analyses (Extended Data Figs. 6 and 7) or with patients in the control arm of the randomized phase 3 study^13^ who received subsequent therapy (Extended Data Fig. 8).

### Early ctDNA reduction correlates with overall survival

On-treatment reductions in ctDNA levels have been previously shown to correlate with clinical outcome in studies involving checkpoint blockade^30,32^. Therefore, we evaluated ctDNA levels at baseline and at weeks 5, 9 and 25 after completion of one, two and six cycles of treatment, respectively. Of the 127 patients in the trial, 118 (93%) had evaluable serum samples, with most (109/118; 92%) found to have detectable ctDNA at any timepoint up to and including week 9 (baseline, week 5, week 9). A total of 99 of these 118 patients (84%) had detectable ctDNA at baseline and on treatment, of whom 94 had mutations detected in one or more uveal melanoma genes (*GNAQ*, *GNA11*, *SF3B1*, *PLCB4*, *CYSLTR2*) at a variant allelic frequency of >0.3 at baseline and were included in the analyses (Methods, Table 1 and Supplementary Table 4). Given that patient data were limited at week 25, this timepoint was excluded from analysis. In the subset of patients (*n* = 45) with both baseline ctDNA data and mutational analysis from tumour biopsies, there was good concordance: 82% of the known uveal melanoma-specific mutations in ctDNA were also found in tumour biopsies; for mutations in *GNAQ/GNA11* the concordance was 85% (Supplementary Table 5), and 43 of 45 tumour samples (96%) had likely loss of one copy of *BAP1* (see Methods). However, the sensitivity of base calling in the sequencing of tumour biopsies was lower due to a lack of matched normal tissue. For patients with known uveal mutations detected in both tumour biopsies and ctDNA (*n* = 38), concordance was 97%. Only one patient had detectable *EIF1AX* mutations in ctDNA at week 5.

**Extended Data Fig. 9:**
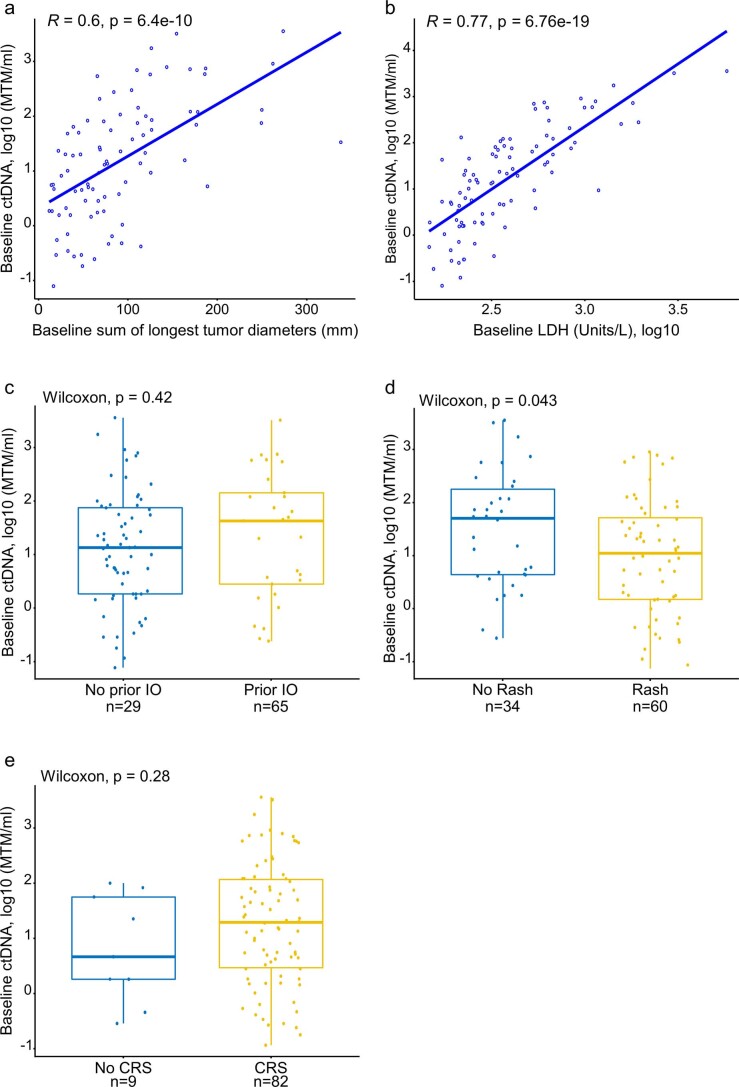
Baseline ctDNA level correlated with tumour burden. **(a-b)** Correlation between level of ctDNA at baseline and (a) tumour size at baseline (n = 94; R = 0.6, p = 6e-10) and (b) LDH level at baseline (n = 91; R = 0.77, p = 6.76e-19). P-value and rho determined using two-sided Spearman rank correlation test. Tumour size was measured as the sum of longest diameters (mm) of the target lesions at first scan according to RECIST v1.1 by Independent Central Review. (c-e) Level of ctDNA at baseline was plotted for **(c)** patients who received prior IO therapy (n = 59) versus patients who did not (n = 35) (p = 0.42), **(d)** patients who experienced Rash in Week 1 (n = 60) versus patients who did not (n = 34) (0.043) and **(e)** patients who experienced CRS (n = 82) versus patients who did not (n = 9) (p = 0.28). Pairwise comparison between groups were conducted using two-sided Wilcoxon signed-rank test. The median is represented by the middle line while the upper and lower borders of the box identify the 75th and 25th percentiles, respectively. The whiskers correspond the minimum and maximal values that is within 1.5 times the interquartile range.

**Extended Data Fig. 10:**
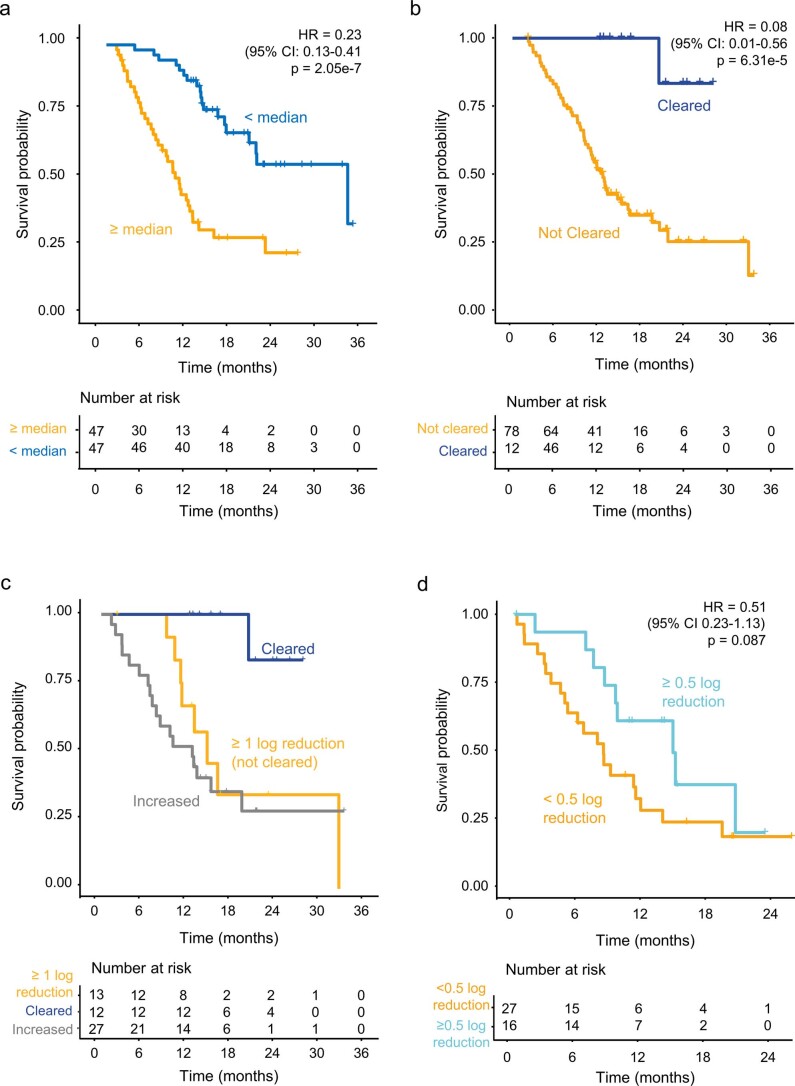
Baseline and on-treatment ctDNA level correlated with overall survival. **(a)** Kaplan–Meier analysis of OS for patients with > median ctDNA levels at baseline versus patients with < median ctDNA levels at baseline (HR 0.23; 95% CI 0.13-0.41, p = 2.05e-7). **(b)** Kaplan–Meier analysis of OS landmarked to week 9 in patients with ctDNA clearance (n = 12) versus patients without clearance (n = 78) by 9 weeks (HR 0.08; 95% CI 0.01-0.56, p = 6.31e-5) and **(c)** Kaplan–Meier analysis of OS in patients with ctDNA clearance (n = 12) versus patients with ≥1 log ctDNA reduction (p = 0.001) and patients with increased ctDNA by 9 weeks (HR 0.74; 95% CI 0.32-1.71, p = 0.476). **(d)** Kaplan–Meier analysis of OS landmarked to week 9 in patients with best overall response of progressive disease with a reduction in ctDNA by ≥ 0.5 log fold change (n = 16) versus < 0.5 log fold change (n = 27) by week 9. Hazard ratio for death 0.51 (95% CI 0.23-1.13), p = 0.087. (a-d) Hazard ratio and confidence intervals were generated from Cox proportional hazard model. P values were generated from two-sided Cox likelihood ratio test.

Mean tumour molecules per ml serum detected at baseline was strongly correlated with tumour burden as defined using the RECIST sum of longest diameters of the target lesions (Spearman’s *r* = 0.6, *P* = 6.4 × 10^−10^) and baseline LDH (Spearman’s *r* = 0.77, *P* = 6.76 × 10^−19^; Extended Data Fig. 9a,b, respectively). Likewise, subgroups with larger tumours or serum alkaline phosphatase or LDH above ULN were more likely to have ctDNA levels above the median (Table 1). No association of baseline ctDNA level with prior immunotherapy was detected (*P* = 0.42; Extended Data Fig. 9c), although a higher percentage of patients who received anti-programmed cell death 1 (anti-PD1) or anti-PD-L1 monotherapy had below-median levels of baseline ctDNA (*P* = 0.019, Fisher’s exact test; Table 1). However, patient numbers are small, and such an association was not observed for anti-cytotoxic T lymphocyte antigen (anti-CTLA) monotherapy or combination anti-PD1 and anti-CTL4. There was also no association between baseline ctDNA level and cytokine release syndrome, but baseline ctDNA was marginally higher in patients who did not have rash in week 1 (Extended Data Fig. 9d,e). Consistent with the association with measures of tumour burden (RECIST sum of longest diameters of the target lesions and LDH), baseline ctDNA levels were associated with overall survival. The subset of patients with below-median levels of ctDNA had longer overall survival compared with the subset with above-median levels of ctDNA at baseline (HR 0.23, 95% CI: 0.13–0.41, *P* = 2.05 × 10^−7^; Extended Data Fig. 10a). Notably, seven of the nine patients without detectable ctDNA at baseline were alive after 12 months and none of the nine patients had detectable ctDNA by week 9.

**Fig. 3:**
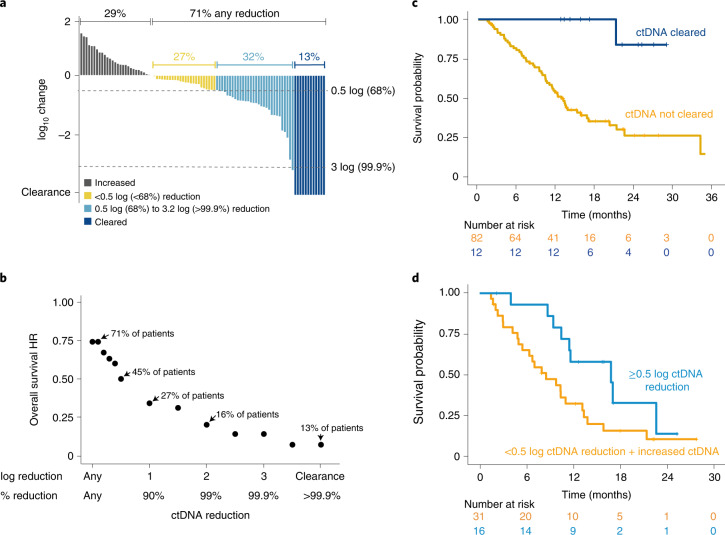
On-treatment ctDNA reduction correlated with survival benefit. **a**, Waterfall plot showing log_10_ change in ctDNA level by week 9 in all evaluable patients (*n* = 94). Percentages have been rounded to the nearest whole number. ﻿**b**, Correlation between ctDNA reduction in patients with metastatic uveal melanoma by week 9 with tebentafusp and the HR for death (*R*^2^ = 0.9, *P* = 8.89 × 10^−7^ by linear model (two sided); *n* = 94). Hazard ratios were derived by comparing subsets of patients with ctDNA above or below the thresholds given on the *x* axis. **c**,**d**, Kaplan–Meier comparison of overall survival in patients with ctDNA clearance (*n* = 12) versus patients without clearance (*n* = 82) by 9 weeks (HR 0.08, 95% CI: 0.01–0.54, *P* = 4.22 × 10^−5^) (**c**) and patients with best overall response of progressive disease with a reduction in ctDNA by ≥0.5 log fold change (*n* = 16) versus <0.5 log fold change (includes patients with increased ctDNA; *n* = 31) by week 9 (HR for death 0.47, 95% CI: 0.22–1.01, *P* = 0.042) (**d**). **b**–**d**, Hazard ratios and confidence intervals were generated using a Cox proportional hazard model. *P* values were generated using a two-sided Cox likelihood ratio test.

By weeks 5 and 9, 66% of patients (59/90) and 71% of patients (67/94), respectively, with baseline and on treatment measurements of mean tumour molecules per ml serum had any (>0) ctDNA reduction (Supplementary Table 6 and Fig. 3a). A total of 29% of patients (27/94) had an increase in ctDNA by week 9, and there were no patients who did not have some change from baseline ctDNA levels (Fig. 3a). Twelve patients had complete ctDNA clearance (undetectable), of whom one had a partial response, seven had stable disease, three had progressive disease and one was non-evaluable by RECIST (Supplementary Table 7). For the remaining 82 patients without ctDNA clearance, three had a partial response, 34 had stable disease, 44 had progressive disease and one was non-evaluable by RECIST (Supplementary Table 7). The magnitude of ctDNA reduction by week 9 was strongly associated with improvement in overall survival (*R*^2^ = 0.9, *P* = 8.89 × 10^−7^): a 0.1 log reduction was associated with an HR of 0.8, while a 1 log reduction was associated with an HR of 0.4, a 2 log reduction was associated with an HR of 0.2, a 3 log reduction was associated with an HR of 0.2, and ctDNA clearance was associated with an HR of 0.1 (Fig. 3b). The 1 year overall survival rate in patients with ctDNA clearance (*n* = 12) was 100% compared with 52% in those with increased ctDNA (*n* = 27) (Fig. 3c and Extended Data Fig. 10b). Even in the subset with ≥1 log reduction in ctDNA but without clearance there was a trend for longer overall survival (Extended Data Fig. 10c). Notably, of the 47 patients with a best radiographic response of progressive disease who were also evaluable for ctDNA, one-third (*n* = 16) had a ≥0.5 log reduction in ctDNA by week 9, including three with ctDNA clearance; 14 patients (30%) had <0.5 log reduction and 17 (36%) had increased ctDNA (Supplementary Table 7). In these patients with progressive disease, ctDNA reduction of ≥0.5 log (including patients who cleared their ctDNA) was associated with improved overall survival, compared with patients with progressive disease with a <0.5 log ctDNA reduction or ctDNA increase (HR 0.47, 95% CI: 0.22–1.01, *P* = 0.042; Fig. 3d and Extended Data Fig. 10d). Of the 16 patients with progressive disease with ≥0.5 log reduction of ctDNA by week 9, 12 developed new lesions.

## Discussion

Data from this study provide the longest follow-up of overall survival and safety of a soluble TCR therapeutic to date. The observed 1 year overall survival rate of 62% and median overall survival of 16.8 months compare very favorably with an analysis using data on a similar population of previously treated patients with metastatic uveal melanoma from a recent meta-analysis that resulted in a 1 year overall survival of 37% and a median overall survival of 7.8 months^18^. These data also support the recent approval of tebentafusp for HLA-A*02:01-positive adult patients with unresectable metastatic uveal melanoma regardless of prior treatment history in the United States, European Union and United Kingdom.

In this cohort of previously treated patients, tebentafusp had a predictable and manageable safety profile supported by a very low rate (3%) of treatment discontinuation due to treatment-related adverse events and no treatment-related deaths. As observed in the phase 1 study and the recent phase 3 study, most treatment-related adverse events recorded could be classified as either skin related or cytokine mediated, consistent with tebentafusp’s proposed mechanism of action. After each of the first three doses patients need to be monitored for at least 16 hours, to facilitate prompt intervention and management when the risk for cytokine release syndrome is highest. Following the first few doses, treatment-related adverse events tend to decrease in both frequency and severity, enabling the monitoring period to be shortened to 30–60 minutes.

RECIST v1.1 underestimated the degree of clinical benefit from tebentafusp. Although the primary endpoint of overall response rate was low at 5%, 44% of patients achieved some degree of tumour shrinkage. More than 70% of all of the patients were treated beyond initial radiographic progression, and nearly half of the patients with tumour growth (≥20%) as best response were alive at 12 months. This pattern of clinical benefit is similar to what was observed in the randomized phase 3 trial of tebentafusp versus investigator’s choice in previously untreated patients with metastatic uveal melanoma, in which tebentafusp-treated patients with a best response of progressive disease had a better overall survival than patients with a best response of progressive disease on the investigator’s choice control arm (HR 0.43)^13^. Together, these data highlight the need for new measures of clinical activity that correlate with overall survival for this new class of immuno-oncology therapy.

Higher levels of ctDNA at baseline have been shown to be correlated with tumour burden and poor prognosis, while on-treatment reductions in ctDNA are associated with improved outcomes, including prolonged progression-free survival and overall survival^30–33,41^. In a recent analysis of samples from patients with 16 advanced stage tumour types treated with checkpoint inhibition (durvalumab ± tremelimumab), early on-treatment reductions in ctDNA were not only associated with improved survival but were also able to be used to differentiate responders from non-responders in patients who had radiologic stable disease at their first assessment^41^. Likewise, in this study we observed a significant linear relationship between the level of ctDNA reduction and overall survival. Baseline ctDNA levels correlated with tumour burden and, by week 9 on tebentafusp, more than two-thirds of patients had some degree of ctDNA reduction, with greater reduction being associated with longer survival. This association was true even for patients with a best radiographic response of progressive disease, three of whom had complete ctDNA clearance, which may reflect changes in tumour size on treatment due to immune activation and infiltration into the tumour rather than frank tumour growth, as proposed for other forms of immuno-oncology therapy^42,43^. Additionally, tumour-infiltrating lymphocytes, edema and tumour necrosis without clearance of debris could also result in the appearance of radiographic progression in the presence of ctDNA reduction or clearance.

In a study of 125 melanoma patients, Lee et al.^34^ demonstrated that ctDNA reduction had utility in identifying the nine patients with pseudoprogression and was associated with overall survival in that subset. In this study with a larger percentage of patients having long overall survival despite apparent radiographic progression, we used a quantitative approach to demonstrate that ctDNA reduction even without complete clearance can provide useful information regarding benefit from tebentafusp. These findings suggest that early reductions in ctDNA reflect tebentafusp-related activity in the tumour and may provide a more precise molecular predictor of clinical response to tebentafusp than traditional radiographic response criteria. Further studies are needed to assess how changes in ctDNA correlate with other pharmacodynamic changes including T cell infiltration into the tumour^44^, and whether other potential surrogate endpoints, such as radiomic features of tumour lesions (particularly change in tumour heterogeneity)^45^, can be identified that better capture these changes^46^.

Commonly used approaches for analyzing ctDNA include next-generation sequencing or digital droplet (dd) polymerase chain reaction (PCR)^47^. Both approaches have their strengths and limitations: ddPCR can be economical but needs to be focused on known mutations and can be challenging to optimize for certain mutations. Next-generation sequencing can enable discovery of new mutations but can be less cost-effective. Here, we used multiplex PCR followed by next-generation sequencing to enable discovery of new variants and for convenience, given that it fitted into other sequencing projects ongoing in the laboratory.

Our study was limited by the absence of a control arm. To interpret the clinical findings, we compared overall survival from this study with that of published studies of first-line ipilimumab plus nivolumab as well as the Rantala et al. and the Khoja et al. meta-analyses^16–18^. We also performed a propensity score analysis that accounts for differences in baseline prognostic factors between patients in this study and patients randomized to the control arm of the phase 3 study (IMCgp100-202) who received subsequent therapy after progression^13^. This latter dataset represents the most recent overall survival follow-up of a second line plus metastatic uveal melanoma population available in the public domain. In each case, the overall survival from previously treated metastatic uveal melanoma for patients who received tebentafusp in this study was found to be superior to the comparator.

The exploratory analysis of ctDNA levels was a retrospective hypothesis-generating analysis using samples collected from a single-arm phase 2 trial, thus limiting interpretation. These results need to be confirmed in a prospective randomized study before ctDNA dynamics on tebentafusp can be introduced to routine clinical practice to manage patient treatment.

In conclusion, tebentafusp demonstrates a promising survival benefit for patients with metastatic disease that has progressed on at least one line of prior therapy, with ctDNA as an early indicator of benefit. The addition of ctDNA analysis to assess the molecular response to treatment may provide a more sensitive means than standard imaging studies to identify those patients who will benefit most from tebentafusp treatment.

## Methods

### Study design and participants

This open-label, international, phase 1/2 study (NCT02570308) was composed of a phase 1 dose escalation and an initial expansion cohort that was subsequently expanded into a full phase 2 expansion study. The primary objective of the phase 1 portion of the study was to identify the maximum tolerated dose and determine the recommended phase 2 dose, the results of which are reported elsewhere^27^. The primary objective of the phase 2 portion was to estimate the objective response rate based on RECIST v1.1 (ref. ^38^) in patients treated at the recommended phase 2 dose of tebentafusp. Secondary objectives included assessment of the safety and antitumour efficacy of tebentafusp with the parameters of overall survival, progression-free survival, disease control rate (defined as the proportion of patients with either an objective response (that is, partial or complete response) or a best overall response of stable disease recorded at least 24 weeks (±1 week) after the date of first dose of study drug), time to response, duration of response (defined as the time from the date of first documented objective response (complete or partial response) until the date of documented disease progression or death by any cause in the absence of disease progression), and the rate and duration of minor response (defined as tumour response with a 10%–29% reduction in the sum of the longest diameters of the target lesions).

Patients with an initial assessment of progressive disease according to RECIST v1.1 could continue therapy beyond initial progressive disease, provided that they did not have symptomatic progression requiring alternative therapy and the investigator believed they were continuing to derive clinical benefit. The modified immune-related RECIST (irRECIST) criteria^39^ were used to evaluate response to treatment beyond progression. Tumour-based endpoints were assessed by a blinded, independent central review with investigator assessment data collected as a secondary evaluation. To assess potential predictors of the efficacy of tebentafusp, change in serum ctDNA level in response to treatment was also measured.

The trial was carried out in accordance with the principles of the Declaration of Helsinki and Good Clinical Practice guidelines, and the study protocol was approved by the relevant ethics bodies at each participating site: Princess Margaret Cancer Centre, Toronto, Canada; Charite Universitaetsmedizin Berlin – Campus Benjamin Franklin, Berlin, Germany; Universitaetsklinikum Heidelberg, Heidelberg, Germany; Institut Catala d’Oncologia (ICO) l’Hospitalet, Hospital Duran i Reynals, Barcelona, Spain; Hospital Universitario Virgen Macarena, Seville, Spain; Centro de Investigación Biomédica en Red de Cáncer (CIBERONC), Madrid, Spain/Hospital Universitario La Paz, Madrid, Spain; Hospital General Universitario de Valencia, Valencia, Spain; The Clatterbridge Cancer Centre, Wirral, UK; Mount Vernon Cancer Centre, Northwood, UK; Columbia University Medical Center, New York, USA; Washington University School of Medicine, St Louis, USA; Thomas Jefferson University Hospital, Philadelphia, USA; Vanderbilt University Medical Center, Nashville, USA; Memorial Sloan Kettering Cancer Center, New York, USA; University of Colorado Cancer Center, Aurora, USA; The Angeles Clinic and Research Institute, a Cedars-Sinai Affiliate, Los Angeles, USA; H. Lee Moffitt Cancer Center and Research Institute, Inc., Tampa, USA; University of California San Diego Moores Cancer Center, La Jolla, USA; California Pacific Medical Center, San Francisco, USA; Baylor Scott & White Health, Dallas, USA; Dean A. McGee Eye Institute, University of Oklahoma, Oklahoma City, USA; Georgetown University – Lombardi Comprehensive Cancer Center, Washington, USA; University of Miami Hospital Clinics/Sylvester Comprehensive Cancer Center, USA; The University of Chicago Medical Center, Chicago, USA; Roswell Park Cancer Institute, Buffalo, USA; and Providence Portland Medical Center, Portland, USA. Patients provided written informed consent before being screened for enrollment. The eligibility criteria for study enrollment included being ≥18 years of age and having a histologically or cytologically confirmed diagnosis of metastatic uveal melanoma, a life expectancy of >3 months as estimated by the investigator, a positive test for HLA-A*02:01 as assessed by central assay, measurable disease according to RECIST v1.1, having disease progression while on one or two prior lines of therapy (including chemotherapy, immunotherapy, or targeted therapy) in the metastatic or advanced setting, and an Eastern Cooperative Oncology Group (ECOG) performance score of ≤1. Patients were excluded from the study if they had symptomatic or untreated central nervous system metastases or central nervous system metastases that required doses of corticosteroids within 3 weeks prior to study day 1, a history of severe hypersensitivity reactions to other biologic drugs or monoclonal antibodies, out-of-range protocol-defined laboratory parameters, or clinically significant cardiac disease or impaired cardiac function.

### Procedures

Tebentafusp was given intravenously using a step-up weekly dosing regimen that was optimized during the phase 1 portion of the study^27^. In this phase 2 portion of the study, all patients received 20 μg on cycle 1 day 1 (C1D1), 30 μg on cycle 1 day 8 (C1D8), and then the recommended phase 2 dose of 68 μg on cycle 1 day 15 (C1D15) and weekly thereafter in cycles of 4 weeks (28 days). Following at least the first three infusions, patients were observed for a minimum of 16 h for monitoring of vital signs and, if necessary, provision of supportive care. After this induction period and provided that no grade 2 or higher hypotension was noted, the observation period for tebentafusp could be reduced to 30–60 min. Treatment continued until confirmation of disease progression as per the modified irRECIST Criteria, intolerable toxicity, investigator decision, or patient withdrawal of consent. The modification to irRECIST was to redefine confirmed immune-related progressive disease as unequivocal progression of non-target lesions and/or new non-measurable disease or an additional 20% increase in tumour burden (that is, the sum of diameters of both the target and measurable new lesions) from the initial progressive disease assessment per RECIST v1.1 rather than from the nadir. An independent data monitoring committee was established to provide oversight of safety and efficacy considerations and to give advice and recommendations regarding steps to ensure both patient safety and the ethical integrity of the study. Radiologic assessments were performed every 8 weeks from C1D1 until C11D1 (40 weeks), then every 12 weeks until progressive disease as per RECIST v1.1, immune-related progressive disease as per the modified irRECIST for patients who continued treatment beyond progressive disease per RECIST v1.1, or discontinuation of study drug.

### Outcomes

Treatment efficacy was assessed using RECIST v1.1 and Kaplan–Meier survival analysis. Overall survival was measured from the start of treatment to the time of death. Patients were censored on the last date on which they were known to be alive. Adverse events were assessed by the investigator and graded as per the National Cancer Institute Common Terminology Criteria for Adverse Events (CTCAE), version 4.03, except for cytokine release syndrome, which was graded according to the 2019 ASTCT Consensus Grading for Cytokine Release Syndrome^40^. Rash is a composite term for a list of skin toxicities of any grade (Supplementary Table 1).

### Circulating tumour DNA analysis

Serum samples collected at baseline and at weeks 5, 9 and 25 on treatment were used to assess the ctDNA level. The analysis focused on mutations up to and including the week 9 on-treatment timepoint given that patient data were sparse at the week 25 timepoint. A custom panel of mutations commonly found in uveal melanoma was designed to assess changes in ctDNA (Natera; Supplementary Data Table 4). Using a panel-based ctDNA approach as in this study, the assessment of BAP1 copy number loss and mutations is very challenging because they can be spread across the gene, without a hotspot. Moreover, genomic studies indicated that BAP1 alterations (copy number loss or mutations) are almost always present in the context of other uveal melanoma mutations, particularly GNAQ or GNA11 (refs. ^48,49^), which are well-covered by the ctDNA panel used here. For this reason, BAP1 was not included in this ctDNA panel. ctDNA was amplified using multiplex PCR and analyzed with next-generation sequencing (performed by Natera Inc). Variants with allele frequencies ≤0.3% at baseline were excluded from the analysis^30^.

### Tumour mutation analysis

All of the tumour biopsies were from sites of metastasis, with the majority of samples coming from liver metastases. Tumour biopsies were analyzed for mutations in GNAQ, GNA11, PLCB4, CYSLTR2, SF3B1 and EIF1AX. DNA libraries were generated from tumour biopsy samples, which were snap frozen, using the Illumina ExomeSeq all exon v6 kit. Paired end fragments of 100 bp in length were sequenced (50 million reads per sample) using the Illumina NovaSeq system. The resulting reads were aligned using BWA-MEM (Burrows–Wheeler aligner – maximal exact match) v0.7.15. Reads were mapped to the GRCh38 primary assembly provided by Ensembl. Duplicate reads were flagged using the MarkDuplicate function of Picard to prevent variant call errors. Somatic variants were called using MuTect2 (GATK Somatic SNVs and INDELs 4.1.6.0).

The BAP1 copy number was estimated using CNVkit v0.9.3 in tumour-only mode with purity correction estimates on BAM files generated from the BWA-MEM alignment step (as detailed above). Separate reference files were used for male and female samples. Copy number loss was defined using a −0.2log_2_ copy ratio cut-off as used in other analyses of metastatic uveal melanoma samples^9^. Samples that had a negative log_2_ copy ratio that was greater than −0.2 were called ‘likely loss’ due to the inherent limitations of metastatic sample collection and the absence of a paired normal tissue.

### Statistical analysis

Approximately 150 patients were to be enrolled, including a minimum of 120 patients for RECIST v1.1 evaluation. With 120 patients and an observed objective response rate of 10% or more, the precision around the estimation of objective response rate was assessed to be 5.3–16.8% using 95% confidence intervals. The primary analysis of the study was conducted after ≥120 evaluable patients had been enrolled and followed for ≥12 months from the start of treatment.

Data were analyzed and reported based on all patient data up to the data cut-off date of 20 March 2020, by which time all patients had at least 1 year of follow-up from the start of treatment. Confidence intervals for objective response rate and related endpoints were calculated using exact methods. Time-to-event endpoints such as overall survival were analyzed graphically using Kaplan–Meier methods and the median and 95% confidence intervals were calculated using the method of Brookmeyer and Crowley. All analyses of efficacy were performed using PROC FREQ, PROC LIFETEST and PROC PHREG in SAS v9.4 or R 4.1.0 for ctDNA analyses.

The analysis set presented here includes all patients who received at least one full or partial dose of tebentafusp (*n* = 127). All safety analyses were performed using the safety analysis set, which also includes all patients who received at least one full or partial dose of tebentafusp (*n* = 127).

Subgroup analyses of best overall response, overall survival and progression-free survival were conducted for a number of covariates relating to line of therapy, prior therapy, best response to prior therapy, prior immuno-oncology checkpoint inhibitors and prior immunotherapy.

Exploratory analyses that compare overall survival results from this study with external overall survival data from the literature used separate Cox proportional hazards models to derive hazard ratios and 95% confidence intervals for each comparison of tebentafusp to the external dataset. An exploratory overall survival analysis compared patients in this study with patients from the control arm of a randomized study in front-line metastatic uveal melanoma who had progressive disease and received subsequent systemic therapy in a second-line setting according to a statistical analysis plan developed before the analysis took place. Given that patient-level data were available for that analysis, a propensity score model and inverse probability of treatment weights were applied to the Kaplan–Meier estimates. The weighting strategy used was the average treatment effect of the treated^50^, whereby tebentafusp patients from this study received a weight of 1.0 and the patients who received other systemic therapies received a weight of *p*_*i*_/(1−*p*_*i*_), where *p*_*i*_ represents the probability of receiving tebentafusp according to the propensity score model for the *i*-th patient. The hazard ratio comparing tebentafusp to the systemic therapy group was derived from a weighted Cox proportional hazards model and the 95% confidence interval was derived using robust sandwich estimation from the weighted Cox model.

For analysis of ctDNA, survival analysis was carried out using the R package survminer v0.4.9, and the Cox likelihood ratio test was used to assess differences between the survival curves. Univariate Cox proportional hazards methods (R package survival v3.2-11) were used to model the prognostic importance of potential predictors of survival. The correlation between hazard ratio and log reduction in ctDNA was assessed using linear regression (R stats package 4.1).

The Spearman test for correlation, Fisher exact test and the Wilcoxon rank sum test were used to assess associations between baseline ctDNA levels and clinically derived patient groupings (tests were two sided and were carried out using R stats package 4.1).

### Reporting summary

Further information on research design is available in the Nature Research Reporting Summary linked to this article.

## Online content

Any methods, additional references, Nature Research reporting summaries, source data, extended data, supplementary information, acknowledgements, peer review information; details of author contributions and competing interests; and statements of data and code availability are available at 10.1038/s41591-022-02015-7.

## Supplementary information

Supplementary Information Supplementary Tables 1–7

Reporting Summary

Supplementary Table 8 Anonymized gene sequencing mutation data of patients for the five uveal associated genes

## Data Availability

Redacted versions of the IMCgp100-102 study protocol and statistical analysis plan are available at ClinicalTrials.gov (NCT02570308). Upon publication, access to pre-existing summary outputs (tables or figures) of trial level data may be granted to qualified academic researchers in the field upon request and approval by the study management committee and subject to appropriate data sharing and transfer agreements. Requesters should submit a proposal including purpose, data format (for example, sas files), hypothesis and specific rationale to info@immunocore.com. To protect the privacy and confidentiality of the patients in this study, sequencing data supporting the ctDNA and tumour mutational analyses have not been made publicly available in a repository. A source data file containing sequencing data from tumour biopsies for the uveal melanoma associated genes is provided as Supplementary information to this manuscript (Supplementary Table 8). Access to de-identified gene limited datasets may be granted to qualified academic researchers 24 months after publication upon request as outlined above for clinical data.

## Extended data

are available for this paper at 10.1038/s41591-022-02015-7.
