# Supplementary material for: Clinical and molecular response to tebentafusp in previously treated patients with metastatic uveal melanoma: a phase 2 trial
Source: Nat Med. 2022 Oct 13;28(11):2364–73. doi: 10.1038/s41591-022-02015-7 (PMC9671803; doi:10.1038/s41591-022-02015-7)
Supplement: Supplementary file 1 — Supplementary Tables 1–7 [file 41591_2022_2015_MOESM1_ESM.pdf]

nature medicine

Article

https://doi.org/10.1038/s41591-022-02015-7

Clinical and molecular response to tebentafusp in previously treated patients with metastatic uveal melanoma: a phase 2 trial

In the format provided by the authors and unedited

Supplementary Information

This appendix is a supplement to: Carvajal RD, Butler MO, Shoushtari A.N., et al. Clinical and molecular response to tebentafusp in previously treated patients with metastatic uveal melanoma

Clinical trial registration number: NCT02570308

Supplemental Tables

Supplementary Table 1: Composite terms for skin toxicity

Skin toxicity group	No. (%) of Patients with Event (N=127)	Skin toxicity group	No. (%) of Patients with Event (N=127)
**Rash**		**Dry skin**	
Blister	2 (2)	Dry skin	52 (41)
Dermatitis acneiform	8 (6)	**Edema**	
Dermatitis allergic	2 (2)	Eyelid oedema	2 (2)
Dermatitis bullous	1 (1)	Periorbital oedema	34 (27)
Palmar-plantar erythrodysaesthesia syndrome	2 (2)	Skin tightness	2 (2)
Papule	1 (1)	Swelling face	2 (2)
Rash	42 (33)	**Other changes**	
Rash erythematous	6 (5)	Alopecia	12 (9)
Rash generalised	30 (24)	Hyperhidrosis	4 (3)
Rash maculo-papular	51 (40)	Night sweats	11 (9)
Rash papular	1 (1)	Skin mass	11 (9)
Rash pruritic	5 (4)		
Skin exfoliation	28 (22)		
Urticaria	2 (2)		
**Pruritus**			
Pain of skin	3 (2)		
Pruritus	87 (69)		
Pruritus generalised	22 (17)		
Eye pruritus	5 (4)		
Skin burning sensation	1 (1)		
**Pigment change**			
Ephelides	2 (2)		
Eyelash discolouration	2 (2)		
Eyelash hypopigmentation	2 (2)		
Hair colour changes	34 (27)		
Skin hyperpigmentation	21 (17)		
Skin hypopigmentation	25 (20)		
Solar lentigo	1 (1)		
Vitiligo	7 (6)		
**Erythema**			
Erythema	22 (17)		
Generalised erythema	20 (16)		
Photosensitivity reaction	2 (2)		

Supplementary Table 2. Treatment-emergent adverse events (≥10%) by system organ class

System organ class/ Preferred term	No. (%) of Patients with Event (N=127)	System organ class/ Preferred term	No. (%) of Patients with Event (N=127)
Number of patients with any TEAE	127 (100%)	Musculoskeletal and connective tissue disorders	91 (72%)
Blood and lymphatic system disorders	24 (19%)	Back pain	41 (32%)
Anaemia	17 (13%)	Arthralgia	33 (26%)
Cardiac disorders	38 (30%)	Myalgia	23 (18%)
Tachycardia	15 (12%)	Pain in extremity	19 (15%)
Eye disorders	68 (54%)	Nervous system disorders	75 (59%)
Periorbital oedema	34 (27%)	Headache	42 (33%)
Gastrointestinal disorders	113 (89%)	Dizziness	21 (17%)
Nausea	86 (68%)	Psychiatric disorders	42 (33%)
Vomiting	52 (41%)	Insomnia	20 (16%)
Abdominal pain	45 (35%)	Anxiety	13 (10%)
Diarrhoea	33 (26%)	Respiratory, thoracic and mediastinal disorders	69 (54%)
Constipation	30 (24%)	Cough	29 (23%)
Abdominal pain upper	26 (21%)	Dyspnoea	24 (19%)
Gastroesophageal reflux disease	16 (13%)	Oropharyngeal pain	13 (10%)
Dyspepsia	15 (12%)	Skin and subcutaneous tissue disorders	122 (96%)
Abdominal distension	14 (11%)	Pruritus	87 (69%)
General disorders	124 (98%)	Dry skin	52 (41%)
Pyrexia	103 (81%)	Rash maculo-papular	51 (40%)
Chills	84 (66%)	Rash	42 (33%)
Fatigue	78 (61%)	Hair colour changes	34 (27%)
Peripheral edema	44 (35%)	Rash generalised	30 (24%)
Influenza like illness	23 (18%)	Skin exfoliation	28 (22%)
Face edema	15 (12%)	Skin hypopigmentation	25 (20%)
Infections and infestations	56 (44%)	Erythema	22 (17%)
Urinary tract infection	16 (13%)	Pruritus generalised	22 (17%)
Nasopharyngitis	13 (10%)	Skin hyperpigmentation	21 (17%)
Investigations	66 (52%)	Generalised erythema	20 (16%)
Aspartate aminotransferase increased	23 (18%)	Vascular disorders	79 (62%)
Alanine aminotransferase increased	19 (15%)	Hypotension	53 (42%)
Weight decreased	20 (16%)	Hypertension	18 (14%)
Blood alkaline phosphatase increased	13 (10%)	Flushing	16 (13%)
Metabolism and nutrition disorders	69 (54%)	Hot flash	14 (11%)
Decreased appetite	32 (25%)		
Hypophosphatemia	14 (11%)		
Hypokalemia	14 (11%)		
Hypomagnesemia	13 (10%)		

MedDRA = Medical Dictionary for Regulatory Activities; N = Total number of patients; no. = number of patients with an observation; PT = preferred term; SOC = system organ class; TEAE = treatment-emergent AE. Patients with multiple TEAEs per SOC or PT are counted only once in each row. Adverse events were coded using MedDRA v22.0.
Treatment-emergent adverse events were defined as any adverse event with a start date from day of first dose of study drug up to 90 days after last dose of study drug or until start of alternative cancer therapy post-treatment discontinuation, whichever occurred first.

Number (%) of patients were sorted alphabetically for SOC and by descending frequency overall for PT. A patient could have had one or more PTs reported under a given SOC.

Supplementary Table 3. Efficacy subgroup analyses

Subgroup	ORR No. (%; 95% CI)	DCR % (no.; 95% CI)		PFS		OS	
		≥16 Weeks	≥24 Weeks	Median mo (95% CI)	6-mo rate (95% CI)	Median mo (95% CI)	1-yr rate (95% CI)
**Age**							
<65 (n=80)	6 (8%; 3-16%)	38% (30; 27-49%)	25% (20; 16-36%)	2.8 (1.9-4.5)	29% (19-39%)	21.2 (14.1-34.2)	68% (56-77%)
≥65 (n=47)	0 (0%; 0-8%)	21% (10; 11-36%)	19% (9; 9-33%)	2.8 (1.8-3.6)	19% (10-31%)	12.8 (7.9-16.8)	51% (36-65%)
**Sex**							
Male (n=63)	2 (3%; 0-11%)	33% (21; 22-46%)	22% (14; 13-35%)	3.1 (2.0-4.0)	23% (13-34%)	13.5 (11.3-17.7)	59% (46-70%)
Female (n=64)	4 (6%; 2-15%)	30% (19; 19-42%)	23% (15; 14-36%)	2.0 (1.9-3.7)	27% (17-39%)	21.3 (12.2-NC)	64% (51-75%)
**ECOG**							
0 (n=89)	3 (3%; 1-10%)	30% (27; 21-41%)	21% (19; 13-31%)	2.8 (2.0-3.6)	23% (15-32%)	17.0 (12.9-34.2)	66% (55-75%)
≥1 (n=38)	3 (8%; 2-21%)	34% (13; 20-51%)	26% (10; 13-43%)	3.5 (1.8-5.5)	30% (16-45%)	13.4 (7.4-22.5)	53% (36-68%)
**Baseline ALC**							
$<1.0 \times 10^9$/L (n=25)	0 (0%; 0-14%)	12% (3; 3-31%)	8% (2; 1-26%)	1.9 (1.8-1.9)	13% (3-29%)	10.3 (6.3-13.4)	42% (22-61%)
$\geq 1.0 \times 10^9$/L (n=102)	6 (6%; 2-12%)	36% (37; 27-46%)	27% (27; 18-36%)	3.6 (2.1-4.0)	28% (20-37%)	21.2 (13.8-NC)	66% (56-75%)
**Baseline LDH**							
≤ULN (n=53)	3 (6%; 1-16%)	38% (20; 25-52%)	32% (17; 20-46%)	3.6 (2.1-5.5)	33% (21-46%)	28.6 (17.7-NC)	86% (73-93%)
>ULN (n=74)	3 (4%; 1-11%)	27% (20; 17-39%)	16% (12; 9-27%)	1.9 (1.8-3.5)	19% (11-29%)	11.0 (7.4-13.1)	45% (33-56%)
**Baseline ALP**							
≤ULN (n=90)	6 (7%; 3-14%)	37% (33; 27-48%)	28% (25; 19-38%)	3.6 (2.0-4.0)	30% (20-39%)	21.3 (16.8-NC)	73% (62-81%)
>ULN (n=37)	0 (0%; 0-10%)	19% (7; 8-35%)	11% (4; 3-25%)	2.0 (1.8-3.0)	14% (5-27%)	8.5 (3.9-12.2)	35% (20-50%)
**Largest target liver metastasis**							
≤3 cm (n=45)	2 (4%; 1-15%)	40% (18; 26-56%)	36% (16; 22-51%)	3.6 (1.9-5.6)	36% (22-49%)	34.2 (17.0-34.2)	75% (60-86%)
>3 - ≤ 8cm (n=50)	2 (4%; 1-14%)	30% (15; 18-45%)	16% (8; 7-29%)	2.9 (1.8-3.8)	21% (11-33%)	13.4 (9.4-16.8)	60% (45-72%)
>8cm (n=17)	1 (6%; 0-29%)	24% (4; 7-50%)	12% (2; 2-36%)	1.9 (1.7-3.8)	12% (2-31%)	9.4 (2.5-11.4)	25% (8-47%)
No liver metastases on target lesions (n=15)	1 (7%; 0-32%)	20% (3; 4-48%)	20% (3; 4-48%)	3.4 (1.9-5.9)	21% (5-45%)	17.7 (6.1-NC)	67% (38-85%)
**Prior therapy**							
Both systemic therapy and ≥1 LDT (n=36)	1 (3%; 0-15%)	33% (12; 19-51%)	28% (10; 14-45%)	2.0 (1.9-4.0)	28% (15-43%)	21.3 (11.6-34.2)	67% (49-80%)
Systemic therapy only (n=70)	5 (7%; 2-16%)	34% (24; 23-47%)	23% (16; 14-34%)	3.6 (1.9-4.0)	27% (17-37%)	14.1 (11.4-NC)	60% (47-71%)
LDT only (n=21)	0 (0%; 0-16%)	19% (4; 5-42%)	14% (3; 3-36%)	2.1 (1.9-3.7)	15% (4-34%)	13.5 (6.3-NC)	59% (35-77%)
**Best response to prior therapy**							
CR/PR (n=18)	2 (11%; 1-35%)	44% (8; 22-69%)	39% (7; 17-64%)	4.6 (1.9-14.7)	44% (20-66%)	NC (11.6-NC)	72% (46-87%)
SD (n=53)	0 (0%; 0-7%)	36% (19; 23-50%)	25% (13; 14-38%)	3.5 (2.0-4.5)	26% (16-39%)	17.7 (12.8-28.6)	67% (52-78%)
PD/NE/NA/missing (n=56)	4 (7%; 2-17%)	23% (13; 13-36%)	16% (9; 8-28%)	1.9 (1.8-3.5)	18% (9-29%)	13.1 (10.3-16.8)	54% (39-66%)
**Prior IO checkpoint inhibitors (CPI)**							
Refractory* to prior CPI (n=61)	4 (7%; 2-16%)	25% (15; 15-37%)	20% (12; 11-32%)	1.9 (1.8-3.6)	20% (11-31%)	14.1 (11.0-17.7)	60% (46-71%)
Relapsed* following prior CPI (n=29)	2 (7%; 1-23%)	52% (15; 33-71%)	38% (11; 21-58%)	5.5 (2.1-13.9)	41% (24-58%)	28.6 (13.4-NC)	76% (56-88%)
No prior CPI (n=37)	0 (0%; 0-10%)	27% (10; 14-44%)	16% (6; 6-32%)	2.1 (1.9-3.6)	19% (9-34%)	12.9 (7.4-NC)	54% (36-69%)
**Prior immunotherapy**							
Yes (n=93)	6 (7%; 2-14%)	33% (31; 24-44%)	25% (23; 16-35%)	2.8 (1.9-3.8)	26% (18-36%)	16.8 (12.8-22.5)	63% (52-72%)
No (n=34)	0 (0%; 0-10%)	27% (9; 13-44%)	18% (6; 7-35%)	2.1 (1.9-3.6)	21% (9-36%)	13.5 (7.0-NC)	59% (40-74%)

* Refractory defined as prior best response of PD; relapsed defined as prior best response of CR, PR or SD

CI = confidence interval, CR = complete response, DCR = disease control rate, IO = immuno-oncology, LDT = liver-directed therapy, mo = months, NA = not applicable, NC = not calculable, NE = not evaluable, ORR = objective response rate, OS = overall survival, PD = progressive disease, PFS = progression-free survival, PR = partial response, SD = stable disease,

Supplementary Table 4. Variant allele frequency detected at baseline for a targeted panel of uveal specific mutations for ctDNA mutation profiling (n=96).

Gene	HGVSp	Frequency No. (%)	Total Frequency (%)	Median VAF (IQR range)	VAF min	VAF max
GNAQ	p.Q209L<br>p.Q209P	9 (9.38)<br>29 (30.21)	40	3.24 (0.57-12.39)<br>7.87 (1.56-22.07)	0.1<br>0.1	29.6<br>31.4
GNA11	p.Q209L	46 (47.92)	48	3.86 (0.91-11.47)	0.0	37.0
SF3B1	p.K700E<br>p.R625L<br>p.R625H<br>p.R625C	3 (3.12)<br>5 (5.21)<br>13 (13.54)<br>10 (10.42)	32	0.94 (0.5-2.88)<br>0.06 (0.02-0.11)<br>8.43 (2.31-11.13)<br>0.63 (0.25-2.19)	0.1<br>0.0<br>0.1<br>0.1	4.8<br>0.2<br>30.4<br>16.2
PLCB4	p.D630Y<br>p.D630V	3 (3.12)<br>1 (1.04)	4	0.07 (0.06-0.07)<br>18.25 (18.25-18.25)	0.1<br>18.2	0.1<br>18.2
CYSLTR2	p.L129Q	3 (3.12)	3	0.91 (0.62-4.74)	0.3	8.6

VAF, variant allele frequency; IQR, interquartile range

Supplementary Table 5. Specific mutations detected in ctDNA and matched tumour biopsies

	ctDNA		Tumour	
**SUBJID**	**Gene**	**HGVSP**	**Gene**	**HVGSP**
1022107	GNA11	p.Q209L	GNA11	p.Q209L
1023119	GNAQ<br>SF3B1	p.Q209P<br>p.R625H	GNAQ<br>ND	p.Q209P<br>ND
1023123	GNA11<br>SF3B1	p.Q209L<br>p.R625H	GNA11<br>SF3B1	p.Q209L<br>p.R625H
1023133	GNAQ<br>SF3B1	p.Q209P<br>p.R625C	GNAQ<br>SF3B1	p.Q209P<br>p.R625C
1024117	GNA11	p.Q209L	GNA11	p.Q209L
1029102	GNA11	p.Q209L	GNA11	p.Q209L
1029104	GNAQ	p.Q209L	GNAQ	p.Q209L
1032105	GNA11	p.Q209L	GNA11	p.Q209L
1043104	SF3B1	p.R625C	SF3B1	p.R625C
1043106	GNAQ	p.Q209L	GNAQ	p.Q209L
1047102	GNA11<br>SF3B1	p.Q209L<br>p.R625H	GNA11<br>SF3B1	p.Q209L<br>p.R625H
1048103	GNA11	p.Q209L	GNA11	p.Q209L
1048106	GNAQ	p.Q209P	GNAQ	p.Q209P
1023113	CYSLTR2	p.L129Q	CYSLTR2	p.L129Q
1023115	CYSLTR2	p.L129Q	CYSLTR2	p.L129Q
1023117	GNAQ<br>SF3B1	p.Q209P<br>p.K700E	GNAQ<br>SF3B1	p.Q209P<br>p.K700E
1023118	GNAQ	p.Q209L	GNAQ	p.Q209L
1022109	GNAQ	p.Q209P	GNAQ	p.Q209P
1023120	GNAQ	p.Q209L	GNAQ	p.Q209L
1023122	PLCB4	p.D630Y	PLCB4	p.D630Y
1023127	GNA11<br>GNAQ	p.Q209L<br>p.Q209L	GNA11<br>ND	p.Q209L<br>ND
1023130	GNAQ	p.Q209P	GNAQ	p.Q209P
1031102	GNA11	p.Q209L	GNA11	p.Q209L
1032103	GNAQ	p.Q209P	GNAQ	p.Q209P
1045102	GNA11	p.Q209L	GNA11	p.Q209L
1043103	GNAQ	p.Q209P	GNAQ	p.Q209P
1028122	GNAQ<br>ND<br>PLCB4	p.Q209L<br>ND<br>p.D630Y	GNAQ<br>GNA11<br>ND	p.Q209L<br>p.Q209L<br>ND
1029110	GNAQ	p.Q209P	GNAQ	p.Q209P
1021124	GNA11	p.Q209L	GNA11	p.Q209L

	ctDNA		Tumour	
**SUBJID**	**Gene**	**HGVSP**	**Gene**	**HVGSP**
1029105	GNAQ<br>SF3B1	p.Q209P<br>p.R625C	GNAQ<br>SF3B1	p.Q209P<br>p.R625C
1021118	GNA11	p.Q209L	GNA11	p.Q209L
1036101	GNAQ<br>SF3B1	p.Q209P<br>p.R625C	GNAQ<br>ND	p.Q209P<br>ND
1023128	GNA11	p.Q209L	GNA11	p.Q209L
1027105	GNA11<br>SF3B1	p.Q209L<br>p.R625C	GNA11<br>SF3B1	p.Q209L<br>p.R625C
1023131	GNAQ	p.Q209L	GNAQ	p.Q209L
1045101	GNAQ<br>SF3B1	p.Q209P<br>p.R625H	GNAQ<br>SF3B1	p.Q209P<br>p.R625H
1048102	GNA11	p.Q209L	GNA11	p.Q209L
1021123	SF3B1<br>ND	p.R625L<br>ND	ND<br>GNAQ	ND<br>p.Q209P
1035102	GNA11	p.Q209L	ND	ND
1043102	SF3B1	p.R625L	ND	ND
1023125	*GNAQ*	*p.Q209P*	*ND*	*ND*
1023126	*GNAQ*	*p.Q209P*	*ND*	*ND*
1028113	GNAQ<br>SF3B1	p.Q209P<br>p.R625H	ND<br>ND	ND<br>ND
1031103	GNAQ	p.Q209P	ND	ND
1052101	GNA11<br>SF3B1	p.Q209L<br>p.R625H	ND<br>ND	ND<br>ND

ND, not detected

Supplementary Table 6. Percentage of patients with on-treatment ctDNA decrease or increase

	Week 5		Week 9	
On-treatment ctDNA change	No. of pts (%) N=90	No. of pts (%) with BOR=PD N=44	No. of pts (%) N=94	No. of pts (%) with BOR=PD N=47
Any ctDNA reduction (>0)	59 (66%)	25 (57%)	67 (71%)	30 (64%)
≥0.5 log ctDNA reduction (includes pts with ctDNA clearance)	37 (41%)	12 (27%)	42 (44%)	16 (34%)
ctDNA clearance	10 (11%)	2 (5%)	12 (13%)	3 (13%)
<0.5 log ctDNA reduction	22 (24%)	13 (30%)	25 (27%)	14 (30%)
ctDNA increase	31 (34%)	19 (43%)	27 (29%)	17 (36%)

Supplementary Table 7. Best overall RECIST response for patients with ctDNA reduction

Best overall response	No. of pts with ≥0.5 log ctDNA reduction (includes pts with ctDNA clearance)	No. of pts with ≥0.5 log ctDNA reduction (excludes pts with ctDNA clearance)	No. of pts with ctDNA clearance	No. of pts without ctDNA clearance	No. of pts with ctDNA increase
Partial Response	2	1	1	3	2
Stable Disease	23	16	7	34	8
Progressive Disease	16	13	3	44	17
Non-Evaluable	1	-	1	1	-
