# Supplementary material for: Clinical and molecular response to tebentafusp in previously treated patients with metastatic uveal melanoma: a phase 2 trial
Source: Nat Med. 2022 Oct 13;28(11):2364–73. doi: 10.1038/s41591-022-02015-7 (PMC9671803; doi:10.1038/s41591-022-02015-7)
Supplement: Supplementary file 2 — Reporting Summary [file 41591_2022_2015_MOESM2_ESM.pdf]

Corresponding author(s): Takami Sato
Last updated by author(s): Aug 8, 2022

Reporting Summary

Nature Research wishes to improve the reproducibility of the work that we publish. This form provides structure for consistency and transparency in reporting. For further information on Nature Research policies, see our Editorial Policies and the Editorial Policy Checklist.

Statistics

For all statistical analyses, confirm that the following items are present in the figure legend, table legend, main text, or Methods section.

n/a	Confirmed	
☐	☒	The exact sample size (*n*) for each experimental group/condition, given as a discrete number and unit of measurement
☐	☒	A statement on whether measurements were taken from distinct samples or whether the same sample was measured repeatedly
☐	☒	The statistical test(s) used AND whether they are one- or two-sided<br>*Only common tests should be described solely by name; describe more complex techniques in the Methods section.*
☒	☐	A description of all covariates tested
☒	☐	A description of any assumptions or corrections, such as tests of normality and adjustment for multiple comparisons
☐	☒	A full description of the statistical parameters including central tendency (e.g. means) or other basic estimates (e.g. regression coefficient) AND variation (e.g. standard deviation) or associated estimates of uncertainty (e.g. confidence intervals)
☐	☒	For null hypothesis testing, the test statistic (e.g. *F*, *t*, *r*) with confidence intervals, effect sizes, degrees of freedom and *P* value noted<br>*Give P values as exact values whenever suitable.*
☒	☐	For Bayesian analysis, information on the choice of priors and Markov chain Monte Carlo settings
☒	☐	For hierarchical and complex designs, identification of the appropriate level for tests and full reporting of outcomes
☒	☐	Estimates of effect sizes (e.g. Cohen's *d*, Pearson's *r*), indicating how they were calculated

Our web collection on statistics for biologists contains articles on many of the points above.

Software and code

Policy information about availability of computer code

Data collection

Clinical data were collected using Oracle InForm version 6.3. ctDNA data were generated by Natera Inc and stored within Microsoft Excel for Microsoft 365 MSO (16.0.14326.20850). Whole exome sequencing data were stored as fastq files. Processed mutation calls were stored within variant calling files.

Data analysis

Statistical analyses of efficacy data were conducted using SAS version 9.4 (all analyses not involving ctDNA). Confidence intervals for ORR and related endpoints were calculated using exact methods from PROC FREQ. Time-to-event endpoints such as OS were analyzed graphically through Kaplan-Meier methods and the median and 95% confidence intervals (CIs) calculated by the method of Brookmeyer and Crowley all using PROC LIFETEST. Hazard ratios and 95% CIs were calculated using a Cox proportional hazards model through PROC PHREG.

ctDNA data were analyzed using R version 4.1.0. Specifically, survival analysis was carried out using Kaplan–Meier curves (survminer package version 0.4.9). The Cox likelihood ratio test was used to assess differences between the survival curves. Univariate Cox proportional-hazards methods were used to model the prognostic importance of ctDNA reduction on survival (R package survival version 3.2-11).
The relationship between hazard ratio of ctDNA reduction was modeled using linear regression (stats package 4.1.0).
A Spearman correlation was used to measure the association between baseline ctDNA and baseline longest sum of tumor diameters/baseline lactate dehydrogenase levels (R stats package 4.1.0).
Pairwise comparison of baseline ctDNA between clinically relevant patient groups were conducted using Wilcoxon signed-rank test (R stats package 4.1.0).
Fisher's exact tests were conducted between categorical patient groups derived by baseline ctDNA and clinical characteristics (R stats package 4.1.0).

Sequenced exome reads were aligned using BWA-MEM (version 0.7.15). Reads were mapped to the GRCh38 primary assembly provided by Ensembl. Duplicate reads were flagged using the MarkDuplicate function of Picard to prevent variant call errors. Somatic variants were called using MuTect2 (GATK Somatic SNVs and INDELs 4.1.6.0).
BAP1 copy number was estimated using CNVkit version (0.9.3) in tumour-only mode with purity correction estimates on BAM files generated

from the BWA-MEM alignment step (as detailed above). Separate reference files were used for male and female samples. Copy number loss was defined using a -0.2 log2 copy ratio cut-off as used in other analyses of metastatic uveal melanoma samples9. Samples which had a negative log2 copy ratio, but greater than -0.2 were called “likely loss” due to the inherent limitations of metastatic sample collection and the absence of a paired normal tissue.

For manuscripts utilizing custom algorithms or software that are central to the research but not yet described in published literature, software must be made available to editors and reviewers. We strongly encourage code deposition in a community repository (e.g. GitHub). See the Nature Research guidelines for submitting code & software for further information.

Data

Policy information about availability of data

All manuscripts must include a data availability statement. This statement should provide the following information, where applicable:

- Accession codes, unique identifiers, or web links for publicly available datasets
- A list of figures that have associated raw data
- A description of any restrictions on data availability

Redacted versions of the IMCgp100-102 study protocol and statistical analysis plan are available at ClinicalTrials.gov (NCT02570308). Upon publication, access to pre-existing summary outputs (tables or figures) of trial level data may be granted to qualified academic researchers in the field upon request and approval by the study management committee and subject to appropriate data sharing and transfer agreements. Requesters should submit a proposal including purpose, data format (e.g., sas files), hypothesis and specific rationale to info@immunocore.com.

To protect the privacy and confidentiality of the patients in this study, sequencing data supporting the ctDNA and tumour mutational analyses have not been made publicly available in a repository. A source data file containing sequencing data from tumour biopsies for the uveal melanoma associated genes is provided as supplemental information to this manuscript (Supplemental Table 8). Access to de-identified gene limited datasets may be granted to qualified academic researchers 24 months following publication upon request as outlined above for clinical data.

Field-specific reporting

Please select the one below that is the best fit for your research. If you are not sure, read the appropriate sections before making your selection.

☒ Life sciences ☐ Behavioural & social sciences ☐ Ecological, evolutionary & environmental sciences

For a reference copy of the document with all sections, see nature.com/documents/nr-reporting-summary-flat.pdf

Life sciences study design

All studies must disclose on these points even when the disclosure is negative.

Sample size	A minimum of 120 patients were to be enrolled for RECIST v1.1 evaluation. The following considerations were used to justify this enrollment goal: With 120 patients and an observed ORR of 10% or more, the precision around estimation of ORR was assessed to be 5.3% to 16.8% by 95% confidence intervals. This study size also provided an adequate number of patients in which to assess the safety and tolerability of the RP2D. For example, for AEs of interest, if 0 AEs were observed in a cohort of 120 patients, there would be 95% confidence that the true event rate is less than 3%.
Data exclusions	Clinical analyses: All patients who received at least one full or partial dose of tebentafusp (n=127) were included in analyses of efficacy and safety.<br>ctDNA analyses: All patient samples collected were assessed for ctDNA level. Samples that failed QC were removed. Patients without a baseline and at least one on-treatment sample were excluded and genes with a baseline variant allele frequency of ≤0.3 were excluded from analyses.
Replication	Replication was not applicable as this was a clinical study with unique patient samples. For ctDNA analysis, all reagents and methods were optimized and validated by Natera.
Randomization	This was a single arm phase 2 study
Blinding	This was a single arm, open label phase 2 study

Reporting for specific materials, systems and methods

We require information from authors about some types of materials, experimental systems and methods used in many studies. Here, indicate whether each material, system or method listed is relevant to your study. If you are not sure if a list item applies to your research, read the appropriate section before selecting a response.

Materials & experimental systems

n/a	Involved in the study
☒	☐ Antibodies
☒	☐ Eukaryotic cell lines
☒	☐ Palaeontology and archaeology
☒	☐ Animals and other organisms
☐	☒ Human research participants
☐	☒ Clinical data
☒	☐ Dual use research of concern

Methods

n/a	Involved in the study
☒	☐ ChIP-seq
☒	☐ Flow cytometry
☒	☐ MRI-based neuroimaging

Human research participants

Policy information about studies involving human research participants

Population characteristics

The eligibility criteria for study enrolment included being ≥18 years of age and having a histologically or cytologically confirmed diagnosis of mUM, a life expectancy of >3 months as estimated by the investigator, a positive test for HLA-A*02:01 as assessed by central assay, measurable disease according to RECIST v1.1, experience of disease progression while on 1 or 2 prior lines of therapy (including chemotherapy, immunotherapy, or targeted therapy) in the metastatic or advanced setting, and an Eastern Cooperative Oncology Group (ECOG) performance score of ≤1. Patients were excluded from the study if they had symptomatic or untreated central nervous system (CNS) metastases or CNS metastases that required doses of corticosteroids within 3 weeks prior to Study Day 1, a history of severe hypersensitivity reactions to other biologic drugs or monoclonal antibodies, out-of-range protocol defined laboratory parameters, or clinically significant cardiac disease or impaired cardiac function. Demographics and characteristics of patients included in our analysis are listed in Table 1 of this manuscript.

Recruitment

26 study centers in 5 countries (Canada, Germany, Spain, United Kingdom, and United States) consented at least 1 patient. The first and last patient were enrolled on 19 Jan 2017 and 21 Mar 2019, respectively. Patients meeting all eligibility criteria were screened, enrolled and treated on study at each participating site. To capture a representative population of patients with previously treated metastatic uveal melanoma who would be eligible for continued treatment of their disease, patients were recruited at 26 centers in 5 countries with the goal of reducing selection bias.

Ethics oversight

Written informed consent was provided by all study participants. The study protocol was approved by each site's Institutional Review Board: Princess Margaret Cancer Centre, Toronto, Canada; Charite Universitaetsmedizin Berlin – Campus Benjamin Franklin, Berlin, Germany; Universitaetsklinikum Heidelberg, Heidelberg, Germany; Institut Catala d'Oncologia (ICO) l'Hospitalet, Hospital Duran i Reynals, Barcelona, Spain; Hospital Universitario Virgen Macarena, Sevilla, Spain; Centro de Investigación Biomédica en Red de Cáncer (CIBERONC), Madrid, Spain/ Hospital Universitario La Paz, Madrid, Spain; Hospital General Universitario de Valencia, Valencia, Spain; The Clatterbridge Cancer Centre, Wirral, UK; Mount Vernon Cancer Centre, Northwood, UK; Columbia University Medical Center, New York, USA; Washington University School of Medicine, St Louis, USA; Thomas Jefferson University Hospital, Phildelphia, USA; Vanderbilt University Medical Center, Nashville, USA; Memorial Sloan-Kettering Cancer Center, New York, USA; University of Colorado Cancer Center, Aurora, USA; The Angeles Clinic and Research Institute, A Cedars-Sinai Affiliate, Los Angeles, USA; H. Lee Moffitt Cancer Center and Research Institute, Inc. Tampa, USA; University of California San Diego Moores Cancer Center, La Jolla, USA; California Pacific Medical Center, San Francisco, USA; Baylor Scott & White Health, Dallas, USA; Dean A. Mcgee Eye Institute, University of Oklahoma, Oklahoma City, USA; Georgetown University - Lombardi Comprehensive Cancer Center, Washington, USA; University of Miami Hospital Clinics/Sylvester Comprehensive Cancer Center, USA; The University of Chicago Medical Center, Chicago, USA; Roswell Park Cancer Institute, Buffalo, USA; Providence Portland Medical Center, Portland, USA.

An independent data monitoring committee (IDMC) was also established to provide oversight of safety and efficacy considerations and give advice and recommendations regarding steps to ensure both patient safety and the ethical integrity of the study.

Note that full information on the approval of the study protocol must also be provided in the manuscript.

Clinical data

Policy information about clinical studies

All manuscripts should comply with the ICMJE guidelines for publication of clinical research and a completed CONSORT checklist must be included with all submissions.

Clinical trial registration

ClinicalTrial.gov, NCT02570308

Study protocol

Information regarding the study protocol can be found at clinicaltrials.gov

Data collection

Data was collected in 26 study centers in 5 countries (Canada, Germany, Spain, United Kingdom, and United States). The first and last patient were enrolled on 19 Jan 2017 and 21 Mar 2019, respectively. Data collection was the responsibility of the clinical study staff at the site under the supervision of the site Investigator. The study eCRF was the primary data collection instrument for the study. The Investigator had to ensure the accuracy, completeness, legibility, and timeliness of the data reported in the CRFs and all other required reports.

Outcomes

The primary objectives of the phase 2 portion of the trial were to estimate the objective response rate (ORR) based on RECIST v1.137

Outcomes

in patients treated at the RP2D of tebentafusp. Secondary objectives included safety as well as assessment of the antitumour efficacy of tebentafusp with the parameters of overall survival (OS), progression-free survival (PFS), disease control rate (DCR), time to response, duration of response (DOR), and the rate and duration of minor response (MR; defined as tumour response with a 10%–29% reduction in the sum of the longest diameters (SLD)). Treatment efficacy was assessed using Response Evaluation Criteria in Solid Tumours (RECIST v1.1) and Kaplan Meier survival analysis. OS was measured from the start of treatment to time of death. Patients were censored on the last date they were known to be alive. Adverse events were assessed by the investigator and graded per the National Cancer Institute Common Terminology Criteria for Adverse Events (CTCAE), version 4.03, except for cytokine release syndrome which was graded according to 2019 ASTCT Consensus Grading for Cytokine Release Syndrome (Lee et al 2019). Rash is a composite term for a list of skin toxicities of any grade (Supplementary Table 1).
